# Synthetic Studies toward Neotatarine

**DOI:** 10.1021/acs.joc.6c01309

**Published:** 2026-08-10

**Authors:** Ankit Yadav, Jin Kun Cha

**Affiliations:** Department of Chemistry, 2954 Wayne State University, Detroit, Michigan 48202, United States

## Abstract

A modular synthesis of a deoxy neotatarine congener is achieved by an intramolecular [4 + 3] oxyallyl cycloaddition of a furan. The synthesis makes use of two cross-coupling reactions of readily available building blocks and is modular, scalable, and amenable to structural modifications.

## Graphical abstract

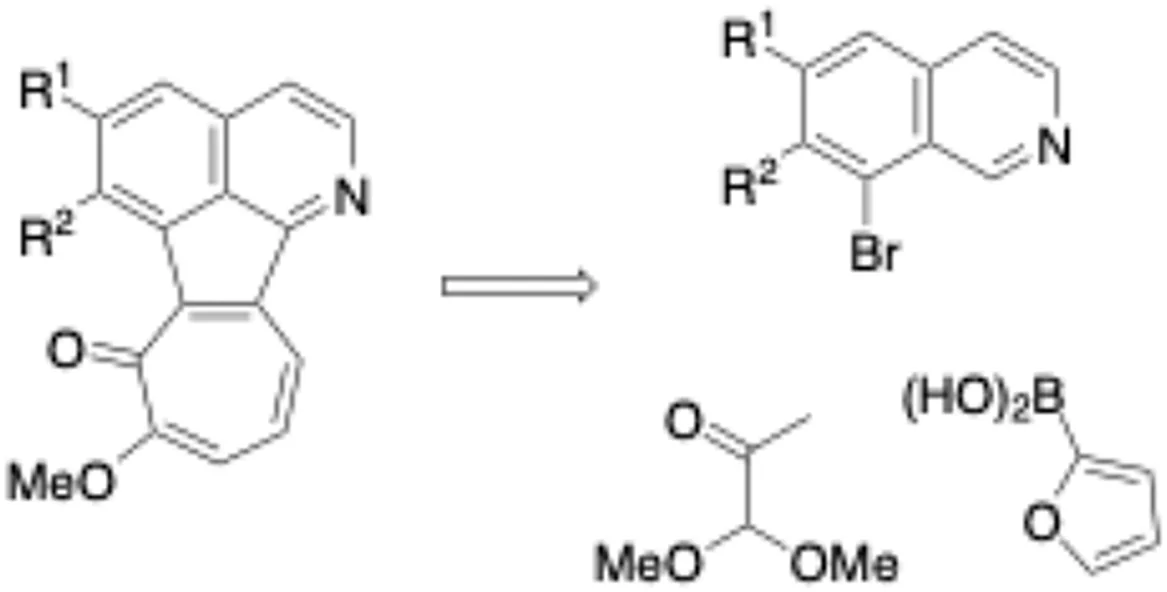

## Introduction

Neotatarine (**1a**) was recently isolated as deep red needles from the genus *Acorus.* It belongs to a small group of the tropoloisoquinoline alkaloids (Figure 1). Cytotoxic properties were reported for these alkaloids, in particular, pareitropone (**2**) and colchicine (**3**), the most prominent tropolone natural product.^2,3^ Interestingly, **1a** appears to lack cytotoxicity, but exhibits significant inhibitory activity against Aβ_25–35_-induced PC12 cell death.^1^ It may thus serve as a new lead in the treatment of neurodegenerative diseases such as Alzheimer’s disease (AD), as ameliorated neuroprotection could mitigate AD progression. What is interesting is whether the placement of the tropolone moiety relative to an isoquinoline subunit in **1a**, which uniquely differs from other members of this family, is the main determinant for biological activity.

**Figure 1:**
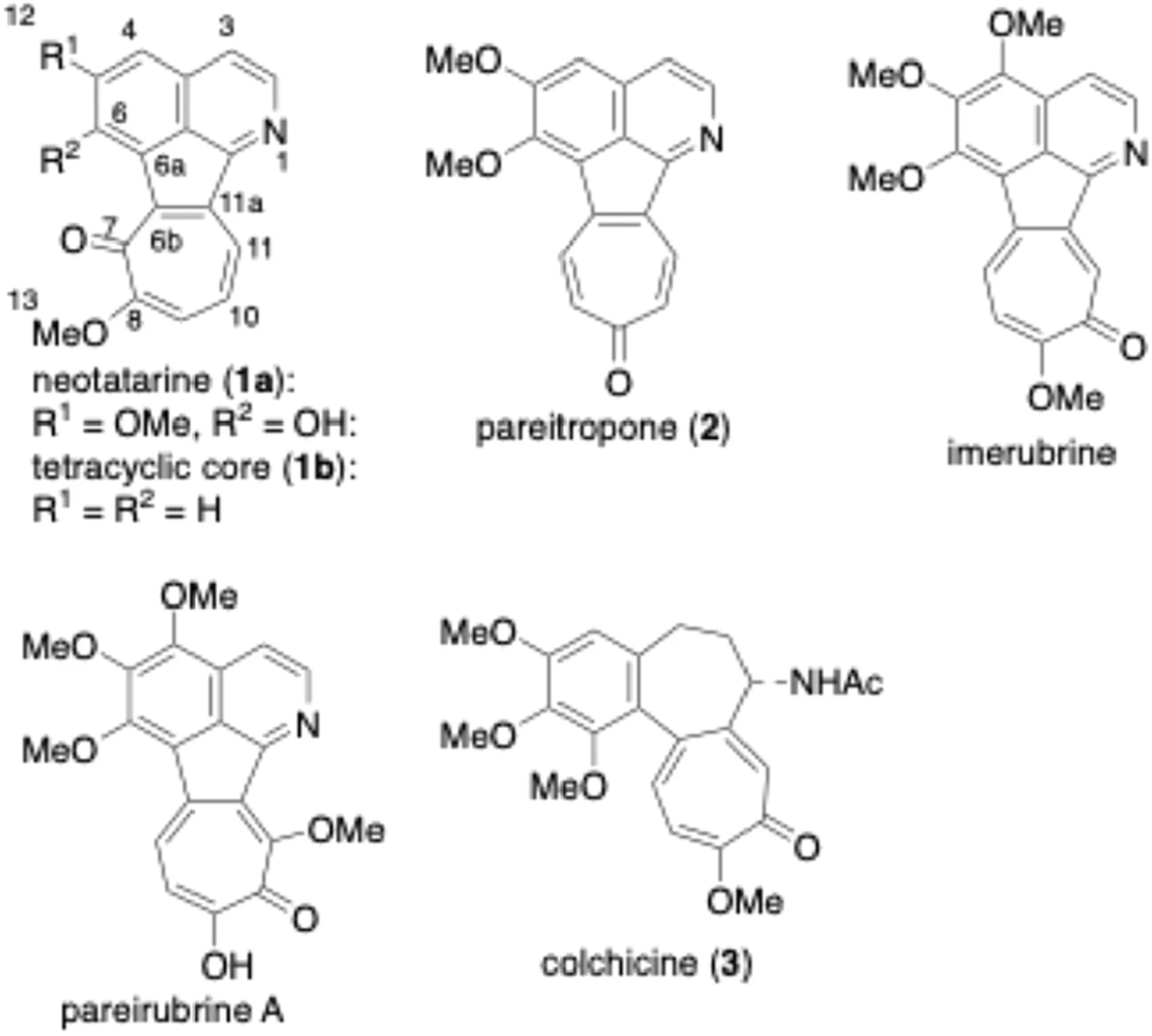
Neotatarine and tropoloisoquinoline alkaloids.

Various methods are available for the synthesis of tropones and tropolones.^4^ Several attractive total syntheses were reported for the tropoloisoquinoline alkaloids, spurred by the unique aromatic heterocycle core and the bioactivity. As part of our continuing interest in this family of natural products,^5,6^ we targeted neotatarine (**1a**) and congeners. An impetus for our synthesis campaign was to undertake a comparative evaluation of the three different approaches to these alkaloids (Figure 2). A successful implementation of ***route A*** is delineated herein:^7a^ an intramolecular oxyallyl [4 + 3] cycloaddition of a furan seemed particularly well suited for the preparation of **1a** and **1b** among the cycloaddition reactions reported for the formation of the requisite ring systems.^4,8,9^ Also advantageous is easy access to the requisite substrate by means of two cross-coupling reactions. We report an expedient synthesis of the neotatarine congener **1b**, which is modular, scalable, and malleable.

**Figure 2:**
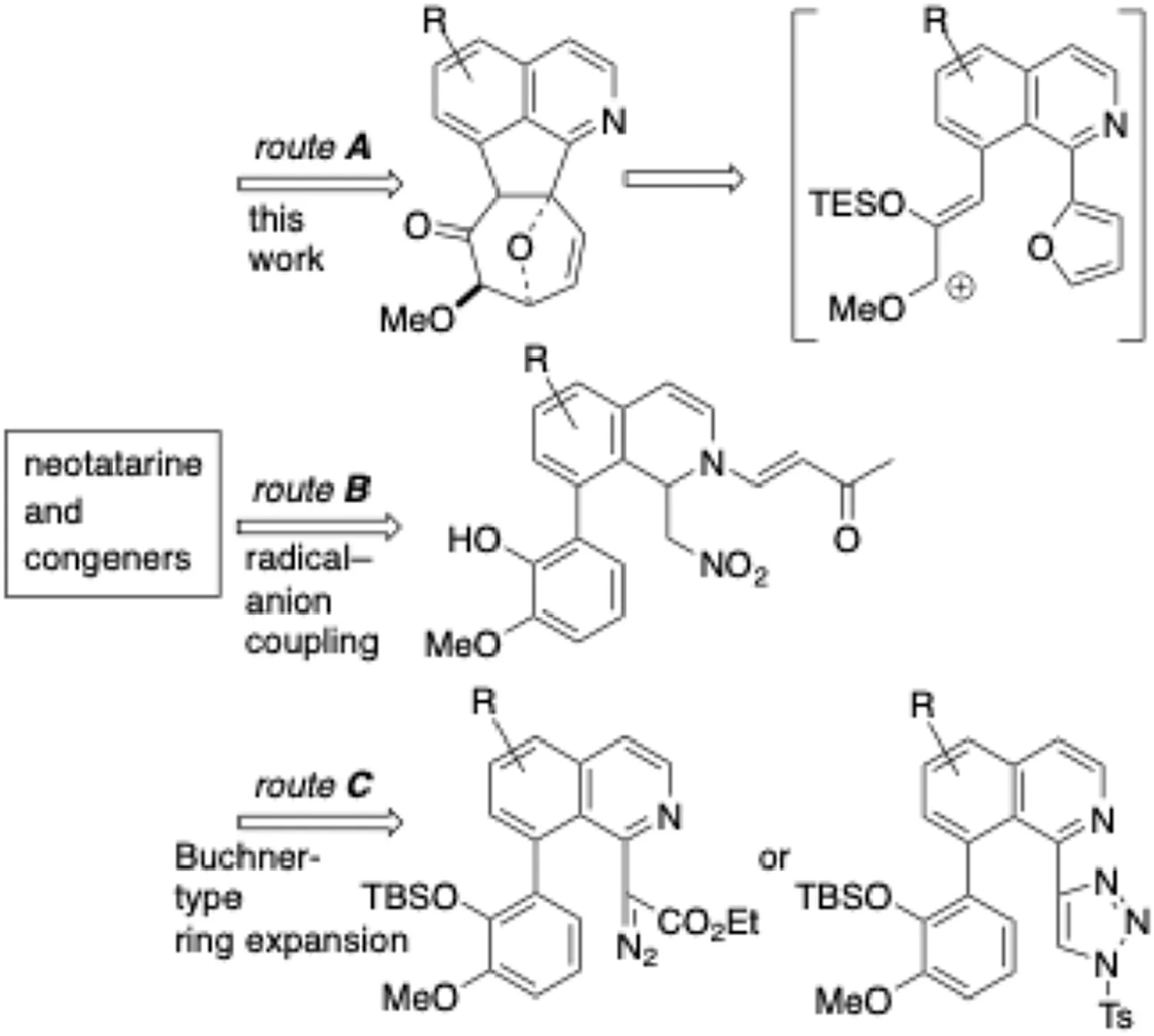
Synthetic approaches to neotatarine.

## Results and Discussion

Our initial studies began with palladium-catalyzed arylation of 8-bromoisoquinolone (**4**)^7b^ with ketone **5** or silylenol ether **6** (Scheme 1). However, **4** proved to be recalcitrant to the Pd-catalyzed coupling reactions, and adaptation of the known procedures to **4** failed to yield **7**.^10–12^ The corresponding enol triflate **8** was prepared and its Suzuki coupling with 2-furanylboronic acid (**9**) afforded **10** chemoselectively in 58% (unoptimized) yield. Unfortunately, the presence of the furan ring was detrimental to the subsequent arylation of **10**: decomposition occurred with **5**, and no reaction was observed with **6**.

**Scheme 1:**
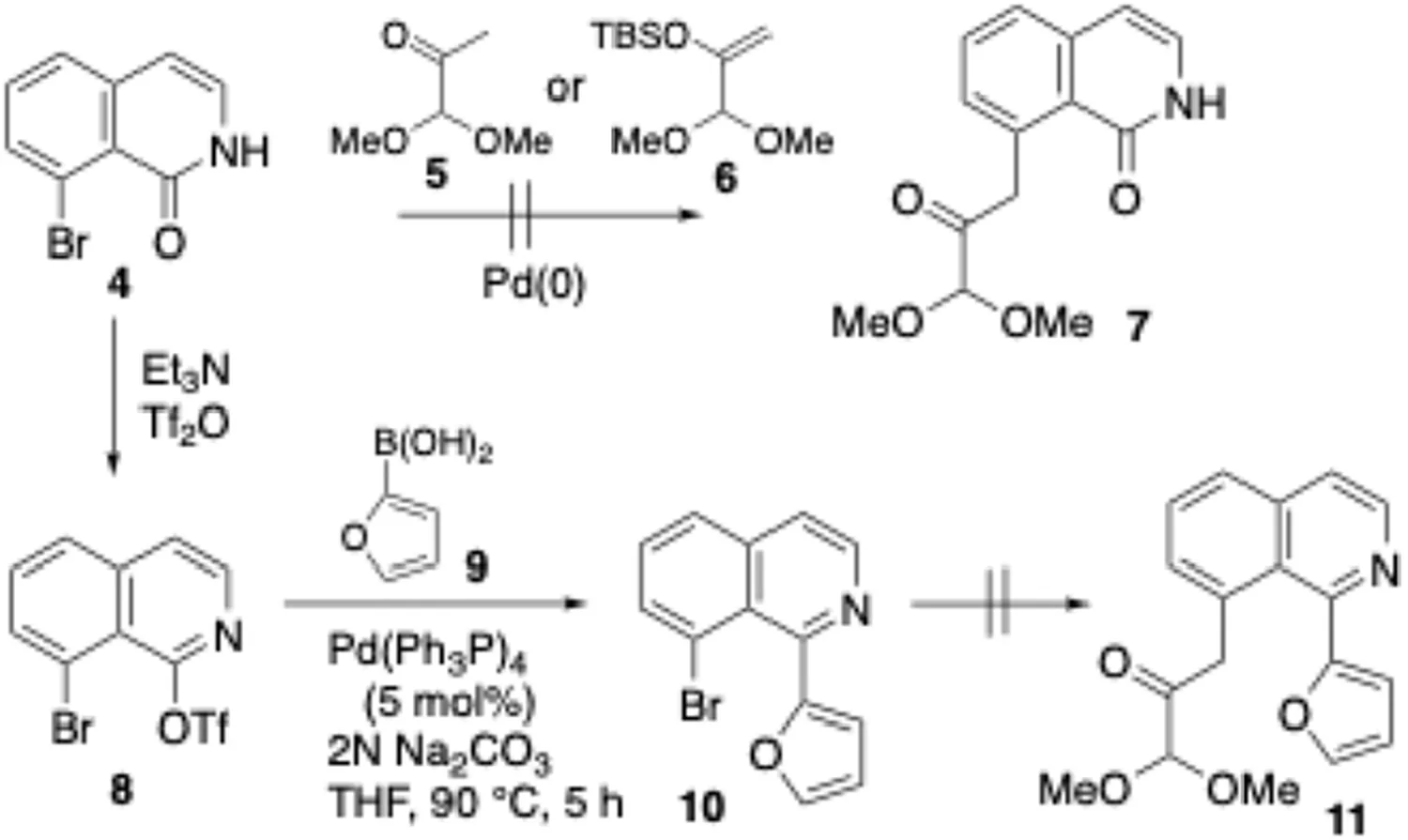
Initial Attempts on the Substrate Preparation

8-Bromoisoquinoline (**12**) was next chosen as starting material (Scheme 2).^7b^ Unlike **4**, clean α-arylation with **5** afforded **13** under Buchwald’s conditions.^11^ Addition of organometallics to in situ generated *N*-acylpyridiniums or *N*-acylisoquinoliniums is well-known,^13^ but would require the keto group be masked. Consequently, these addition reactions were not pursued. The intermediacy of the corresponding *N*-oxide seemed more expedient. Oxidation of **13** to *N*-oxide **14** was achieved by MTO/H_2_O_2_ oxidation.^14b^ *N*-oxide **15** was obtained cleanly (93%) with *m*-CPBA.^14^ Pd-catalyzed α-arylation of **5** with **15** furnished **14** in 81% yield.^11^ Both sequences were equally effective.

**Scheme 2:**
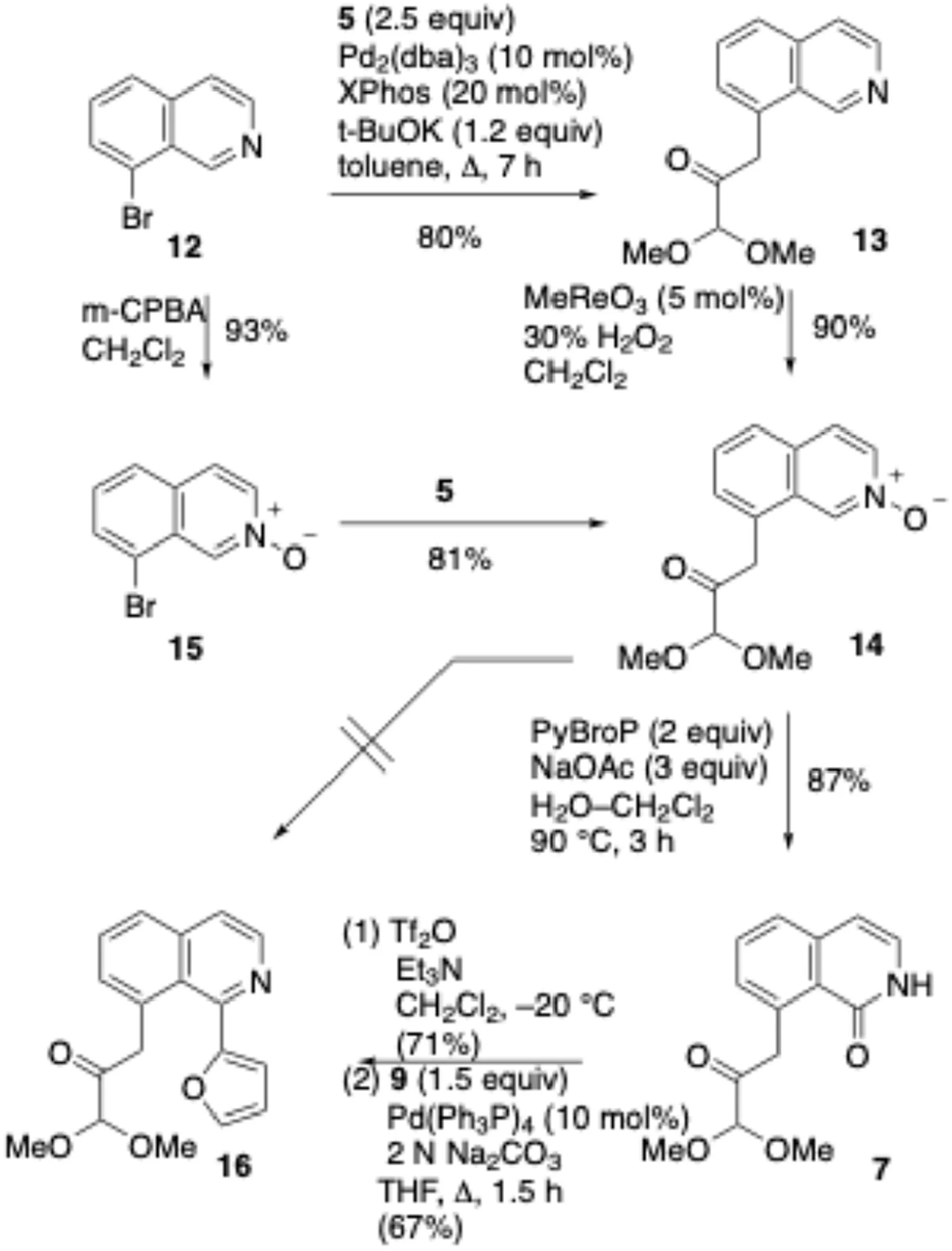
Preparation of the Cycloaddition Substrate

Direct arylation of pyridine and azine *N*-oxides with aryl bromides, catalyzed by Pd­(OAc)_2_ and t-Bu_3_P,^15^ or with aryl boronic esters by the action of Cu­(acac)_2_ and t-BuOK,^16^ was recently developed. However, all these attempts with **14** uniformly failed to produce **16**. Among several activating agents employed for the Reissert-Henze reaction of pyridine and azine *N*-oxides, phosphonium salt PyBroP was particularly effective under mild conditions: activation of **14** with PyBroP in the presence of NaOAc and water delivered quinolone **7** in 87% yield.^17^ Finally, subsequent formation of the enol triflate, followed by the Suzuki coupling with **9**, afforded the requisite coupling product **16** to set the stage for the [4 + 3] cycloaddition.

With **16** in hand, formation of its silylenol ether was the next step (Scheme 3). Although **13** afforded the corresponding silylenol ether smoothly under standard conditions (TBSOTf, Et_3_N, CH_2_Cl_2_), no silylation of **16** was observed under identical conditions. A simple solution was found in the use of a stronger base (t-BuOK, Et_3_SiCl, CH_2_Cl_2_) to provide **17** in 78% yield as the *Z*-isomer. The pivotal [4 + 3] cycloaddition of an α-methoxy-substituted oxyallyl required considerable experimentation:^5,8,18^ typical treatment of **17** with TMSOTf and Et_3_N in nitropropane or methylene chloride yielded predominantly the corresponding keto aldehyde, the trapping product of the oxyallyl cation by adventitious water. A spate of the [4 + 3] oxyallyl cycloaddition reactions notwithstanding, it was surprising that there were only a handful of intramolecular examples.^19^ Ultimately, *inverse* addition afforded the desired [4 + 3] cycloadduct **18**, which was obtained as a single diastereomer in 68% yield. The characteristic coupling constants of ∼ 0 Hz for the pseudoaxial proton (H_8_) at 4.17 ppm and 2.0 Hz (doublet) for the bridgehead proton (H_9_) at 5.22 ppm were indicative of the compact, endolike transition state.^18^ Finally, the tetracyclic core **1b** was secured cleanly (75%) by oxa-bridge cleavage of **18** with excess of TMSOTf.

**Scheme 3:**
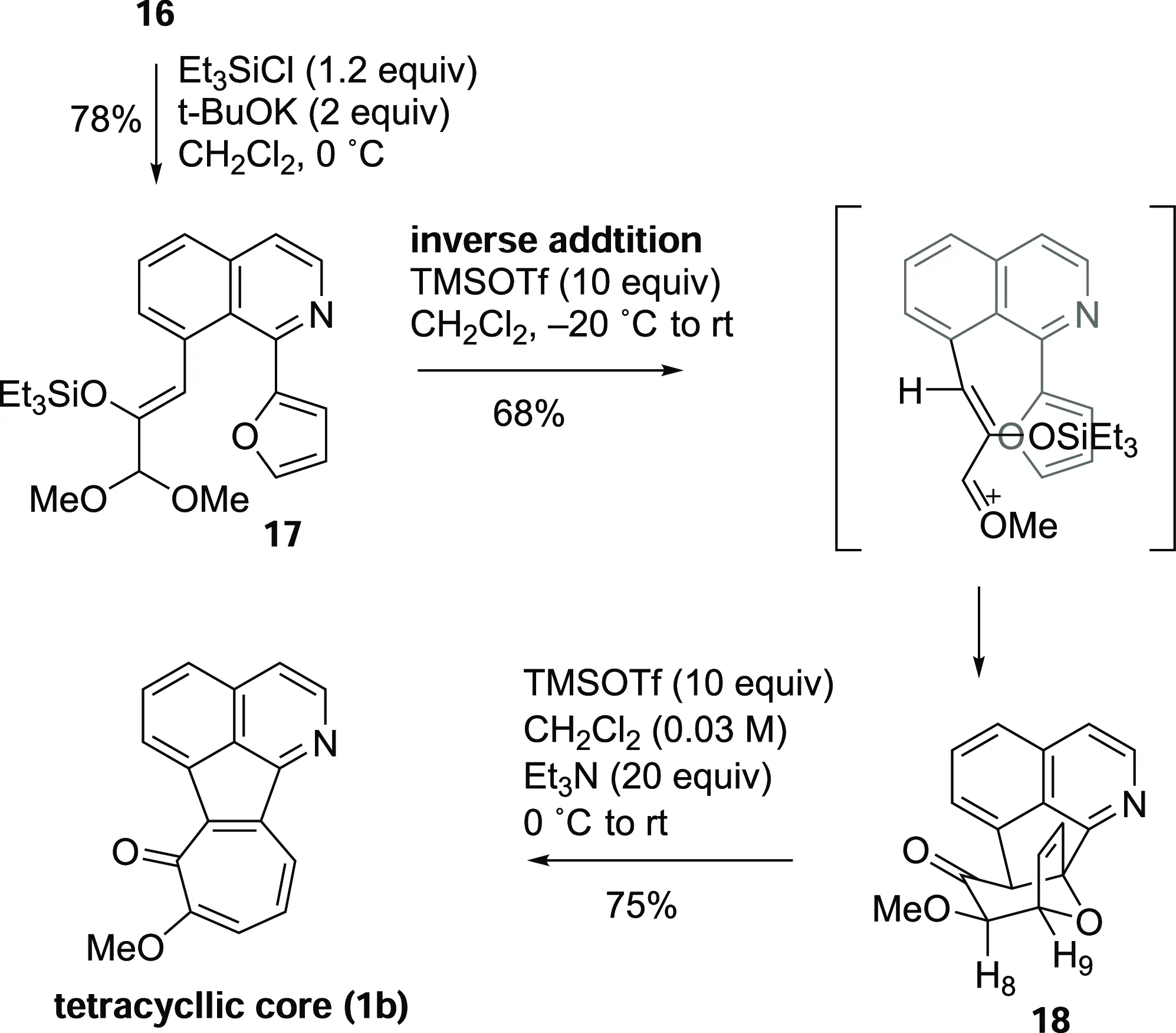
Oxyallyl [4 + 3] Cycloaddition and Ring Opening

## Conclusion

In conclusion, a concise synthesis of the dideoxy congener **1b** of neotatarine is achieved by an intramolecular [4 + 3] oxyallyl cycloaddition of a furan. This work highlights the synthetic utility of intramolecular [4 + 3] oxyallyl cycloaddition reactions in the assembly of the fused tropolones. A modular synthesis is made possible by two cross-coupling reactions (arylation and Suzuki coupling) of three readily available building blocks. The synthesis is expedient, efficient, and scalable. Total synthesis of neotatarine (**1a**) is in progress by adaptation of the identical sequence and will be reported in due course.

## Experimental Section

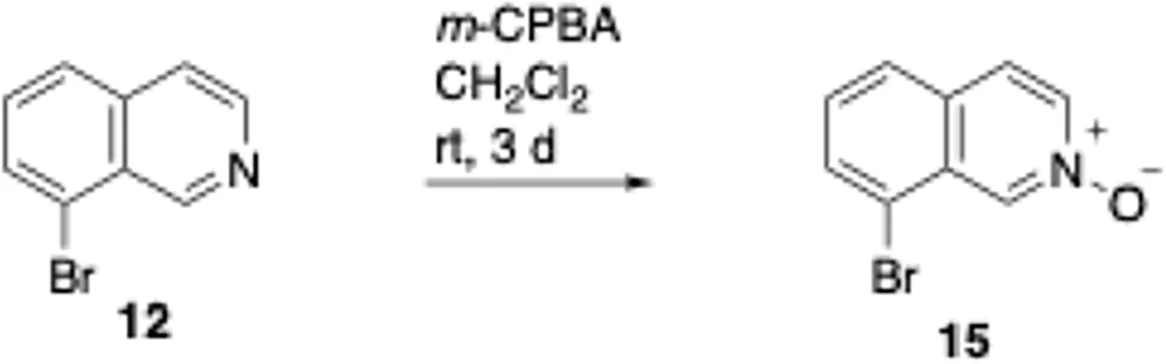



To a solution of 8-bromoisoquinoline (**12**) (1.0 g, 4.8 mmol) in CH_2_Cl_2_ (20 mL) was added *m*-CPBA (2.47 g, 14.4 mmol). The reaction mixture was stirred at rt for 3 days. After the reaction was complete (checked by TLC), it was quenched with saturated aq NaHCO_3_ (10 mL) and extracted with CH_2_Cl_2_ (5 × 5 mL). The combined organic extracts were dried over Na_2_SO_4_ and concentrated in vacuo. The crude material was purified by silica gel column chromatography (9:1 EtOAc/MeOH; *R*_f_ = 0.4) to provide 8-bromoisoquinoline *N*-oxide (**15**) (1.0 g, 93%) as a yellow solid: ^1^H NMR (600 MHz, CDCl_3_) δ 9.04 (s, 1H), 8.10 (d, *J* = 7.0 Hz, 1H), 7.78 (d, *J* = 7.5 Hz, 1H), 7.69 (d, *J* = 8.1 Hz, 1H), 7.61 (d, *J* = 7.0 Hz, 1H), 7.36 (m, 1H); ^13^C­{^1^H} NMR (150 MHz, CDCl_3_) δ 137.7, 136.5, 133.5, 130.1, 129.3, 129.1, 126.4, 124.5, 119.1.
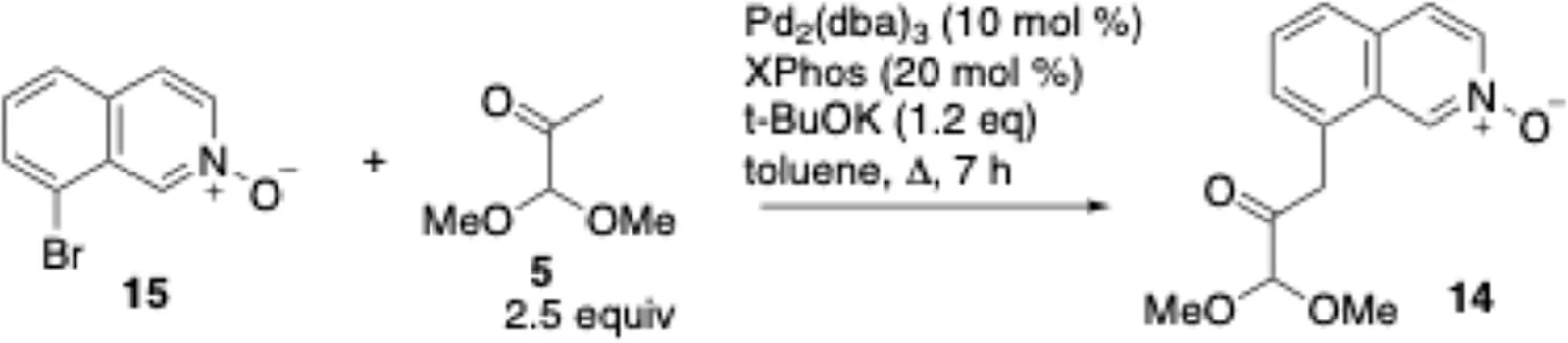


An oven-dried RB flask was charged with **15** (1.0 g, 4.46 mmol), Pd_2_(dba)_3_ (0.44 g, 10 mol %) XPhos (0.424 g, 20 mol %) and t-BuOK (0.6 g, 5.35 mmol). The mixture was evacuated by a vacuum pump and backfilled with argon. The evacuation and backfilling process was repeated. Anhydrous toluene (30 mL) was added, followed by 1,1-dimethoxypropan-2-one (**5**) (1.4 mL, 11.15 mmol). The reaction mixture was heated (in an oil bath) at 120 °C for 7 h. When the reaction was complete, the mixture was allowed to cool to rt, filtered through a pad of Celite, and eluted with EtOAc and hexane, and concentrated in vacuo. The crude material was purified by silica gel chromatography (9:1 EtOAc/MeOH; *R*_f_ = 0.4) to give **14** (952 mg, 81%) as a waxy dark brown solid: IR (neat) 2920, 2850, 1735, 1017 cm^–1^; ^1^H NMR (600 MHz, CDCl_3_) δ 9.28 (s, 1H), 8.51 (d, *J* = 5.7 Hz, 1H), 7.71 (d, *J* = 8.2 Hz, 1H), 7.61 (d, *J* = 5.7 Hz, 1H), 7.56 (m, 1H), 7.38 (d, *J* = 7.0 Hz, 1H), 4.54 (s, 1H), 4.39 (s, 2H), 3.47 (s, 6H); ^13^C­{^1^H} NMR (151 MHz, CDCl_3_) δ 201.8, 149.2, 142.9, 136.4, 131.4, 130.0, 129.8, 127.4, 126.4, 120.9, 104.7, 55.4, 40.3; HRMS (SI): [M + H] 262.1070 calcd for C_14_H_16_NO_4_, found 262.1074.
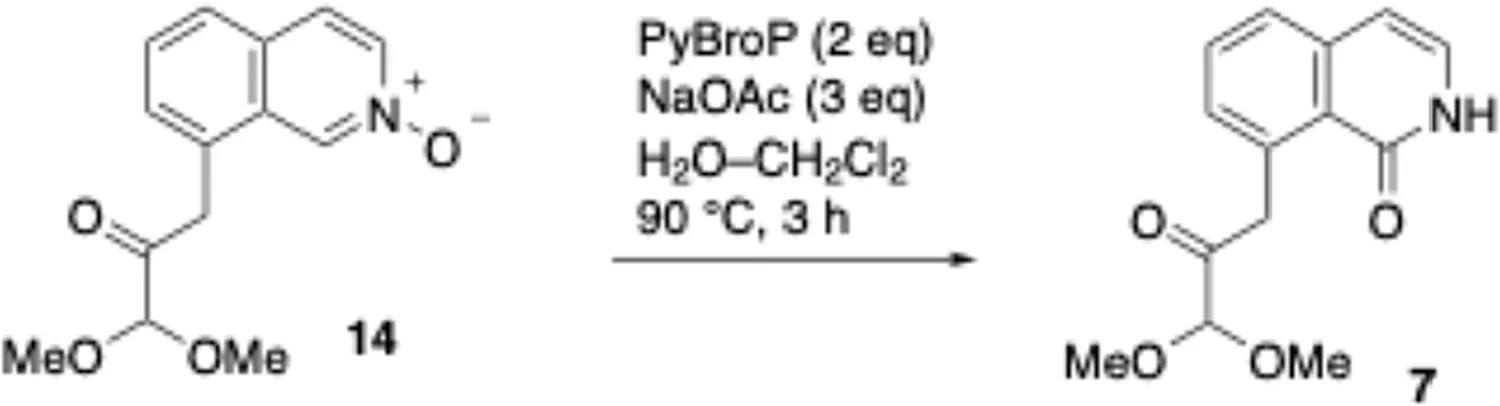


To a stirred solution of **14** (0.74 g, 2.835 mmol) in 1,2-dichloroethane (25 mL) were added at rt sequentially sodium acetate (0.7 g, 8.5 mmol), bromotripyrrolidinophosphonium hexafluorophosphate (2.64 g, 5.67 mmol), and H_2_O (1.3 mL). The reaction mixture was heated (in an oil bath) at 90 °C for 3 h. The mixture was cooled to rt, and saturated aq NaHCO_3_ (8 mL) was added. The mixture was then extracted with ethyl acetate (5 × 10 mL). The combined organic extracts were dried over sodium sulfate and concentrated under vacuo. The crude product was purified by silica gel column chromatography (4:1 Hexane/EtOAc; *R*_f_ = 0.5) to provide **7** (0.65 g, 87%) as a white solid: IR (neat) 1733, 1655, 1638 cm^–1^; ^1^H NMR (600 MHz, CDCl_3_) δ 10.67 (s, 1H), 7.55 (m, 1H), 7.46 (d, *J* = 7.9 Hz, 1H), 7.18 (d, *J* = 7.0 Hz, 1H), 7.07 (m, 1H), 6.50 (d, *J* = 7.0 Hz, 1H), 5.08 (s, 1H), 4.40 (s, 2H), 3.50 (s, 6H); ^13^C­{^1^H} NMR (151 MHz, CDCl_3_) δ 201.5, 164.5, 140.1, 136.8, 132.2, 131.2, 127.7, 126.2, 124.0, 107.4, 103.4, 54.4, 46.3; HRMS (SI): [M + H] 262.1074 calcd for C_14_H_16_NO_4_, found 262.1074.
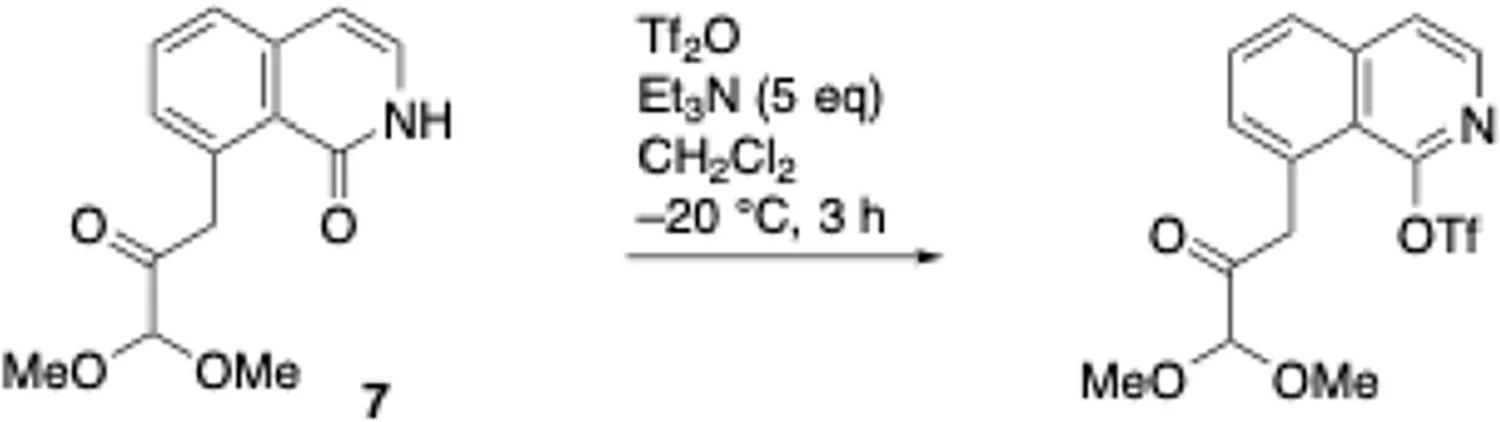


Isoquinolone **7** (160 mg, 0.61 mmol) was dissolved in anhydrous dichloromethane (60 mL). Triflic anhydride (0.36 mL, 2.14 mmol) was added slowly at −20 °C, followed by dropwise addition of *i*Pr_2_EtN (0.53 mL, 3.06 mmol). The resulting mixture was stirred for 3 h. After the reaction was complete, (checked by TLC), the reaction was quenched with saturated aqueous NaHCO_3_ (10 mL) and extracted with CH_2_Cl_2_ (3 × 10 mL). The combined organic extracts were dried over sodium sulfate and concentrated under vacuo. The crude material was purified by silica gel column chromatography (4:1 Hexane/EtOAc; R_*f*_ = 0.8) to provide the corresponding triflate (0.17 g, 71%) as a waxy yellow solid: IR (neat) 1736, 1398, 1213 cm^–1^; ^1^H NMR (600 MHz, CDCl_3_) δ 7.67 (t, *J* = 7.7 Hz, 1H), 7.44–7.43 (m, 2H), 7.27 (d, *J* = 7.5 Hz, 1H), 6.58 (d, *J* = 7.7 Hz, 1H), 4.85 (s, 1H), 4.42 (s, 2H), 3.50 (s, 6H); ^13^C­{^1^H} NMR (125 MHz, CDCl_3_) δ 201.7, 161.2, 139.0, 138.0, 134.6, 133.2, 126.7, 124.8, 123.9, 119.3 (q, *J* = 325 Hz), 110.2, 104.0, 54.8, 45.6; ^19^F NMR (565 MHz, CDCl_3_) δ – 70.
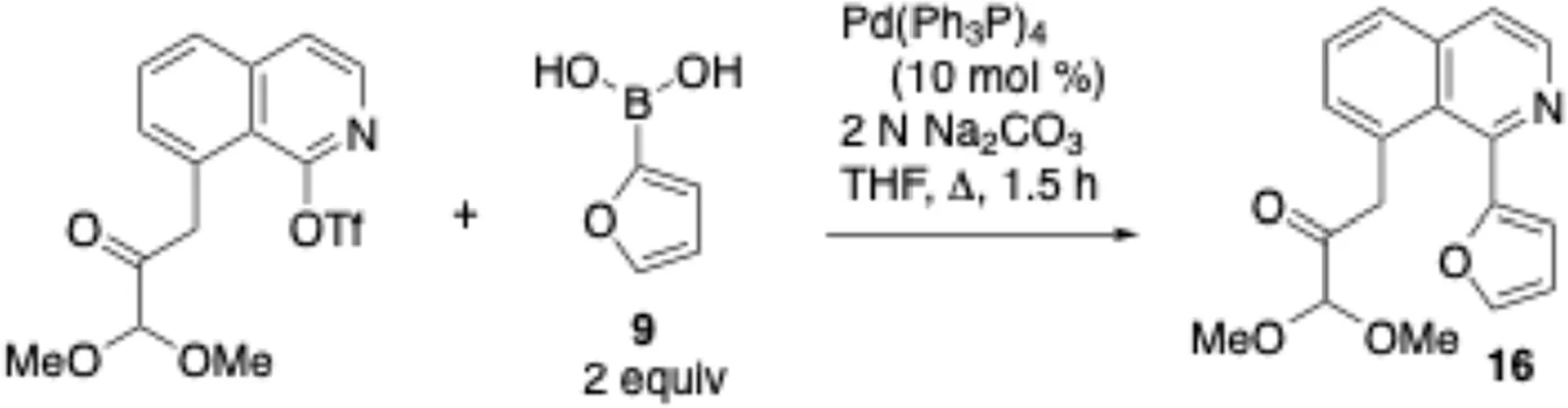


To a solution of the aforementioned triflate (120 mg, 0.3 mmol) in anhydrous THF (10 mL) under a N_2_ atmosphere were added furan-2-ylboronic acid (**9**) (670 mg, 0.61 mmol) and Pd­(PPh_3_)_4_ (35 mg, 10 mol %). When the mixture was dissolved completely, 2 N Na_2_CO_3_ (1.2 mL) was added. The resulting mixture was refluxed (in an oil bath) at 120 °C for 1.5 h. After the reaction was complete (checked by TLC), the mixture was cooled to rt, quenched with water (10 mL), and extracted with ethyl acetate (3 × 5 mL). The combined organic extracts were dried over Na_2_SO_4_ and concentrated in vacuo. Purification by column chromatography (Hexane/EtOAc: 7:3; *R*_f_ = 0.5) afforded **16** (63 mg, 67%) as a pale-yellow oil: IR (neat) 1734, 1070 cm^–1^; ^1^H NMR (600 MHz, CDCl_3_) δ 8.55 (d, *J* = 5.6 Hz, 1H), 7.82 (d, *J* = 7.2 Hz, 1H), 7.70 (d, *J* = 5.6 Hz, 1H), 7.63 (t, *J* = 7.2 Hz 1H), 7.58 (d, *J* = 1.8 Hz, 1H), 7.35 (d, *J* = 7.2 Hz, 1H), 6.72 (d, *J* = 3.3 Hz, 1H), 6.60 (dd, *J* = 3.3, 1.8 Hz, 1H), 4.31 (s, 1H), 3.85 (s, 2H), 3.33 (s, 6H); ^13^C­{^1^H} NMR (151 MHz, CDCl_3_) δ 202.1, 143.0, 141.1, 138.4, 132.4, 132.2, 132.1, 131.5, 129.9, 128.7, 128.6, 127.3, 127.1, 122.0, 111.9, 110.7, 103.1, 54.6, 43.8; HRMS (SI): [M + H] 312.1230 calcd for C_18_H_18_NO_4_, found 312.1231.
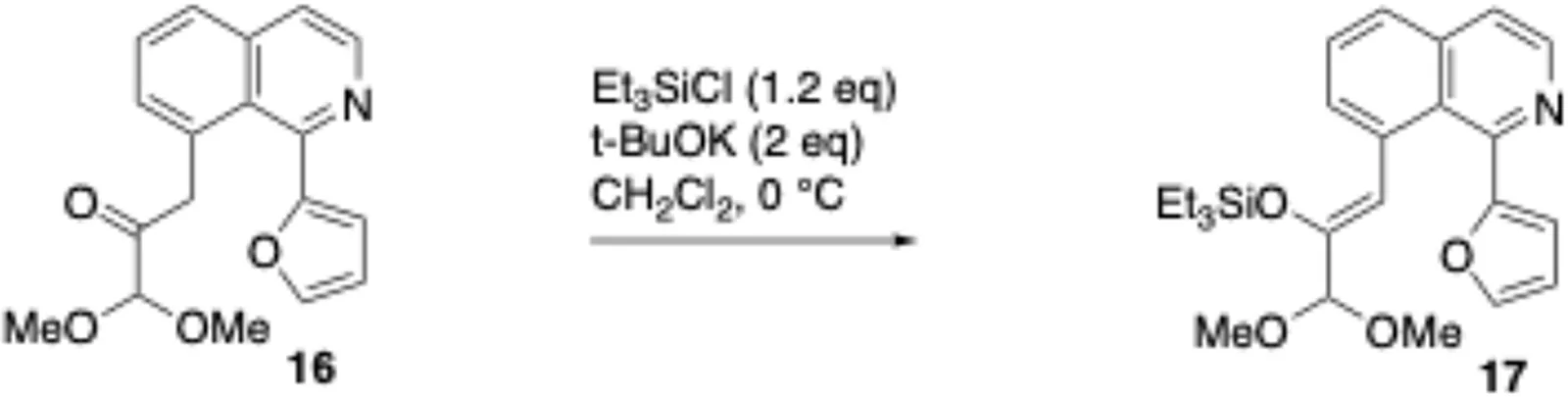


To a stirred solution of **16** (20 mg, 0.064 mmol) in anhydrous dichloromethane (2 mL) under a N_2_ atmosphere were added sequentially triethylsilyl chloride (14 μL, 0.076 mmol) and potassium tert-butoxide (15 mg, 0.128 mmol) at 0 to 10 °C. The reaction mixture was stirred for 1.5 h. After the conversion was completed (checked by TLC), the mixture was concentrated under vacuo. Purification by column chromatography (3:2 Hexane/EtOAc; *R*_f_ = 0.8) afforded **17** (21 mg, 78%) as a yellow oily compound: ^1^H NMR (400 MHz, CDCl_3_) δ 8.52 (d, *J* = 5.5 Hz, 1H), 8.00 (d, *J* = 7.2 Hz, 1H), 7.71 (d, *J* = 7.0 Hz, 1H), 7.62 (m, 1H), 7.60 (d, *J* = 5.5 Hz, 1H), 7.52 (t, *J* = 1.8 Hz, 1H), 6.72 (d, *J* = 3.4 Hz, 1H), 6.54 (dd, *J* = 3.4, 1.8 Hz, 1H), 5.73 (s, 1H), 4.37 (s, 1H), 3.28 (s, 6H), 0.81 (t, *J* = 8.0 Hz, 9H), 0.50 (q, *J* = 8.0 Hz, 6H); ^13^C­{^1^H} NMR (101 MHz, CDCl_3_) δ 146.4, 142.6, 141.1, 138.1, 133.8, 130.4, 129.7, 125.6, 121.3, 111.4, 110.2, 109.0, 103.2, 53.5, 6.7, 5.4.
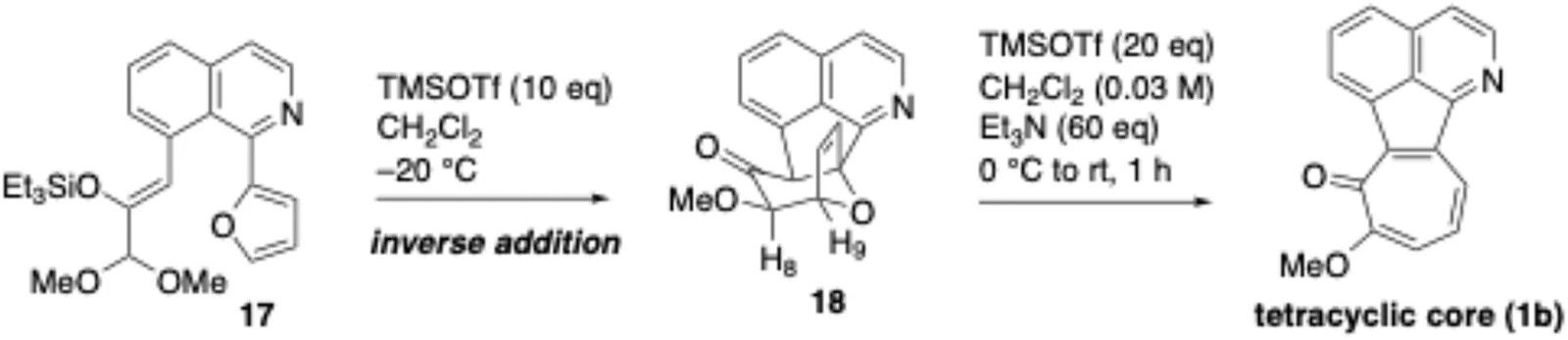


To a stirred solution of TMSOTf (128 μL, 0.704 mmol) in dichloromethane (1 mL) at −20 °C was added dropwise a solution of **17** (30 mg, 0.070 mmol) in dichloromethane (1 mL). The reaction mixture was stirred for 1 h. After the conversion was complete (checked by TLC), the reaction was quenched with aqueous NaHCO_3_ (5 mL) at the same temperature. The mixture was extracted with dichloromethane (3 × 5 mL). The combined organic extracts were dried over Na_2_SO_4_ and concentrated in vacuo. Purification by column chromatography (7:3 EtOAc/Hexane; *R*_f_ = 0.4) afforded **18** (13.4 mg, 68%): IR (neat) 1731, 1621, 1583, 1099, 1044 cm^–1^; ^1^H NMR (400 MHz, CDCl_3_) δ 8.63 (d, *J* = 5.9 Hz, 1H), 7.82 (m, 1H), 7.80 (d, *J* = 6.9 Hz, 1H), 7.75 (d, *J* = 6.8 Hz, 1H), 7.63 (d, *J* = 5.9 Hz, 1H), 6.81 (d, *J* = 5.9 Hz, 1H), 6.77 (dd, *J* = 5.9, 2.0 Hz, 1H), 5.21 (m, 1H), 4.17 (d, *J* = 1.0 Hz, 1H), 3.95 (s, 1H), 3.55 (s, 3H); ^13^C­{^1^H} NMR (101 MHz, CDCl_3_) δ 202.5, 162.3, 146.1, 137.6, 135.2, 135.0, 134.3, 133.4, 133.0, 125.0, 123.0, 119.0, 93.2, 83.9, 83.2, 62.9, 57.9; HRMS (ESI): [M + H] 280.0968 calcd for C_17_H_14_NO_3_, found 280.0968.

To a stirred solution of **18** (10 mg, 0.0358 mmol) in dichloromethane (0.5 mL) at 0 °C was added dropwise TMSOTf (0.13 mL, 0.716 mmol), followed by triethylamine (0.3 mL, 2.14 mmol). The reaction mixture was stirred for 1 h. After the conversion was completed (checked by TLC), the reaction mixture quenched with aqueous NaHCO_3_ (5 mL) and extracted with dichloromethane (3 × 5 mL). The combined organic extracts were dried over Na_2_SO_4_ and concentrated in vacuo. Purification by column chromatography (100% EtOAc, *R*_f_ = 0.3) afforded **1b** (7 mg, 75%): IR (neat) 1580, 1267, 1220 cm^–1^; ^1^H NMR (400 MHz, CD_3_OD) δ 8.66 (d, *J* = 5.7 Hz, 1H), 8.56 (d, *J* = 6.7 Hz, 1H), 7.88 (d, *J* = 10 Hz, 1H), 7.84 (d, *J* = 7.7 Hz, 1H), 7.77 (d, *J* = 5.7 Hz, 1H), 7.73 (m, 1H), 7.44 (t, *J* = 10 Hz, 1H), 7.18 (d, *J* = 10 Hz, 1H), 4.06 (s, 1H); ^13^C­{^1^H} NMR (101 MHz, CD_3_OD) δ 177.3, 167.4, 147.4, 145.8, 140.8, 139.3, 135.8, 134.8, 133.3, 131.7, 128.9, 125.1, 123.3, 123.2, 122.6, 115.5, 57.2; HRMS (ESI): [M + H] 262.0863 calcd for C_17_H_12_NO_2_, found 262.0864.

## Supplementary Material



## Data Availability

The data underlying this study are available in the published article and its Supporting Information.

## Notes

Characterization data for all new compounds (PDF)
