# Supplementary material for: Synthetic Studies toward Neotatarine
Source: J Org Chem. 2026 Aug 10;91(33):11464–8. doi: 10.1021/acs.joc.6c01309 (PMC13505295; doi:10.1021/acs.joc.6c01309)
Supplement: Supplementary file 1 [file jo6c01309_si_001.pdf]

SUPPORTING INFORMATION

Synthetic Studies toward Neotatarine

Ankit Yadav and Jin Kun Cha*

Department of Chemistry, Wayne State University, Detroit Michigan 48202, USA

Corresponding Author:
ao1929@wayne.edu

Table of Contents:

General Information S2

^{1}H and ^{13}C NMR Spectra S3 – S19

1. General Information:

Unless otherwise stated, reactions were performed in oven-dried glassware under a N_2 atmosphere using anhydrous solvents. All glassware, syringes, needles, and magnetic stirring bars used in moisture-sensitive reactions were oven-dried and stored in desiccators prior to use. Moisture- or oxygen-sensitive reactions were conducted under an atmosphere of N_2. Anhydrous toluene, 1,2-dichloroethae ($ClCH_2CH_2Cl$), dichloromethane (CH_2Cl_2), and tetrahydrofuran (THF) were purchased from Fisher. Deuterated chloroform ($CDCl_3$) was purchased from Cambridge Isotope Laboratories, Inc. and was used without further purification. Reaction temperatures were controlled by an immersion temperature modulator. Thin-layer chromatography (TLC) was performed using silica gel 60 F254 precoated plates (0.25 mm) and visualize by UV. NMR spectra were measured on commercially available spectrometers (^1H at 400 & 600 or ^{13}C at 151 & 100 MHz) in $CDCl_3$ or CD_3OD. Tetramethylsilane was used as an internal standard. Unless otherwise noted, all compounds purified by chromatography were sufficiently pure by ^1H NMR analysis.

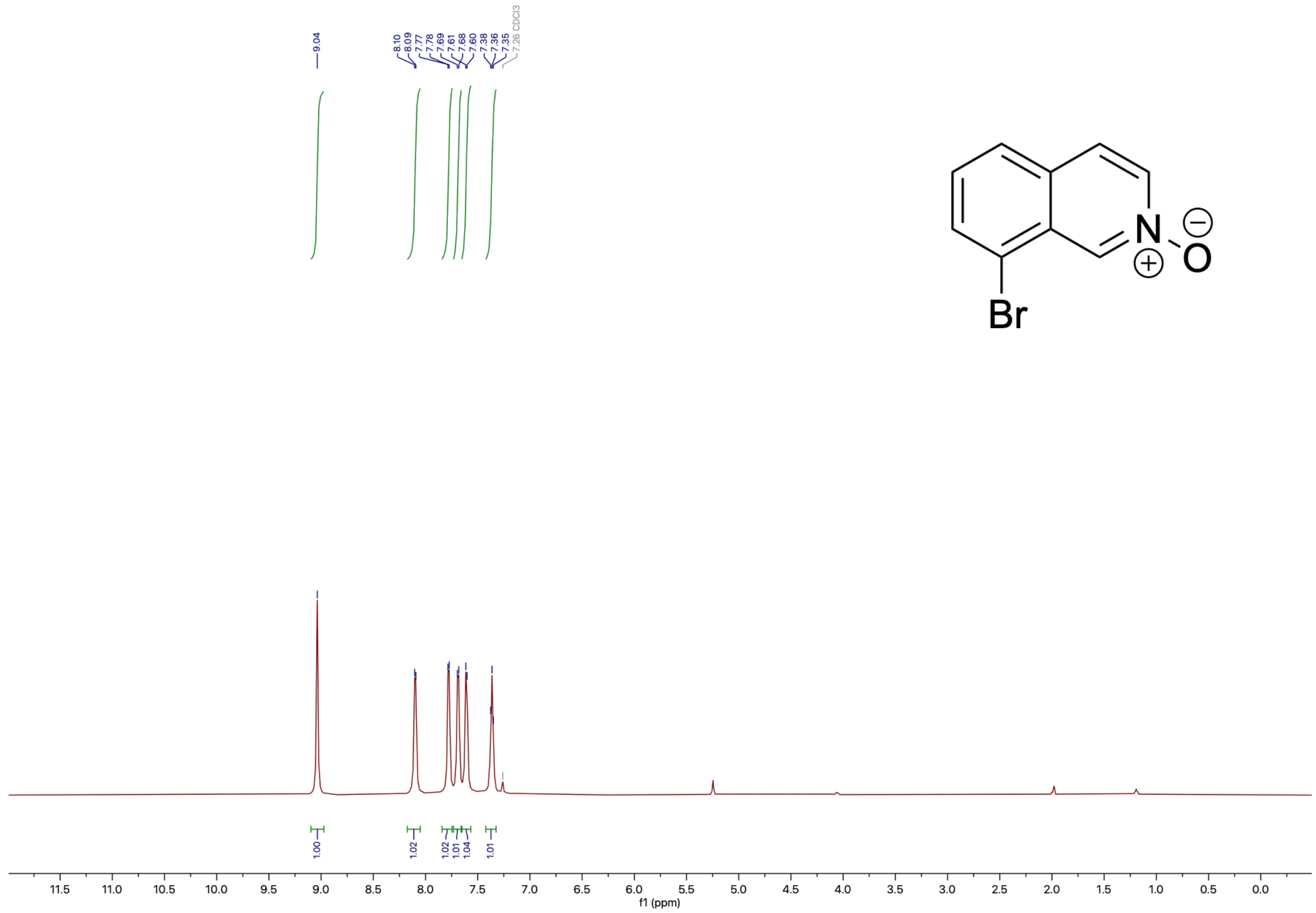

^{1}H NMR Spectrum (600 MHz, $CDCl_3$) of **15**

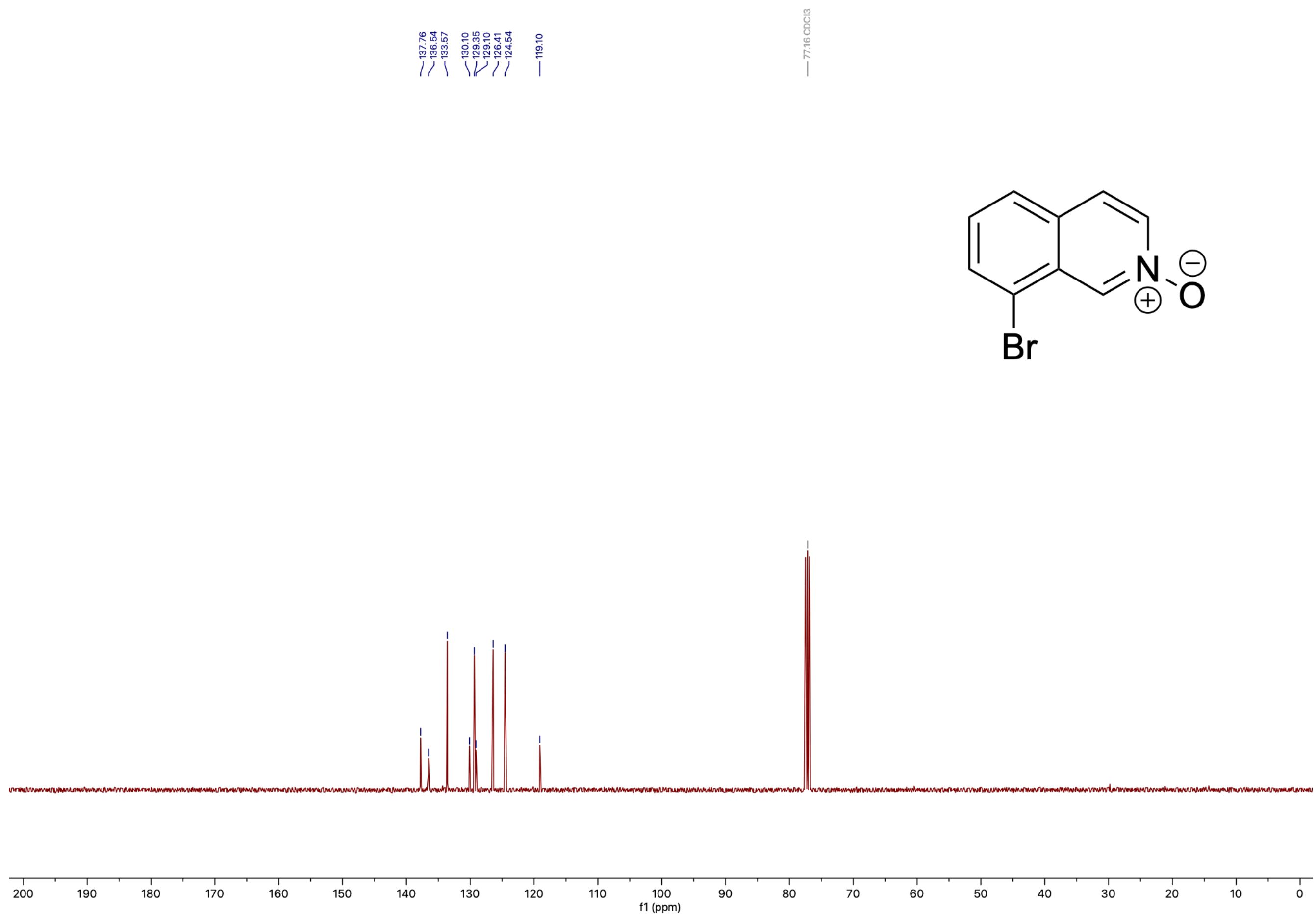

$^{13}C\{^{1}H\}$ NMR Spectrum (151 MHz, $CDCl_3$) of **15**

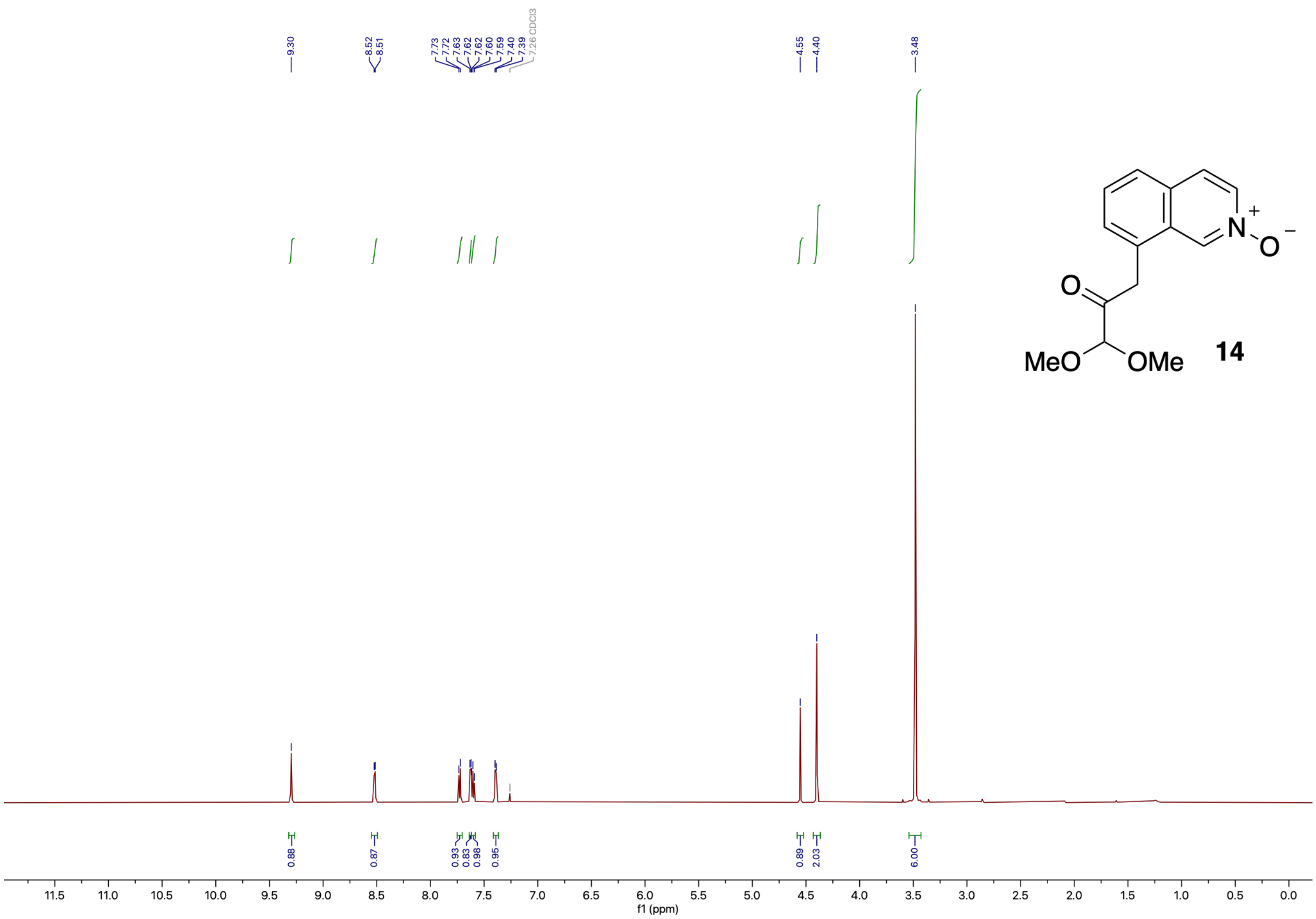

^{1}H NMR Spectrum (600 MHz, $CDCl_3$) of **14**

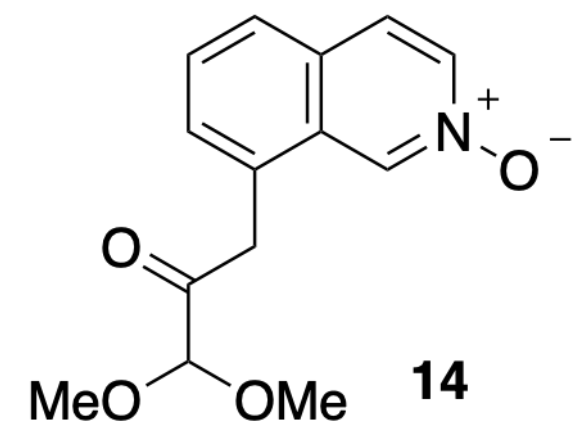

14

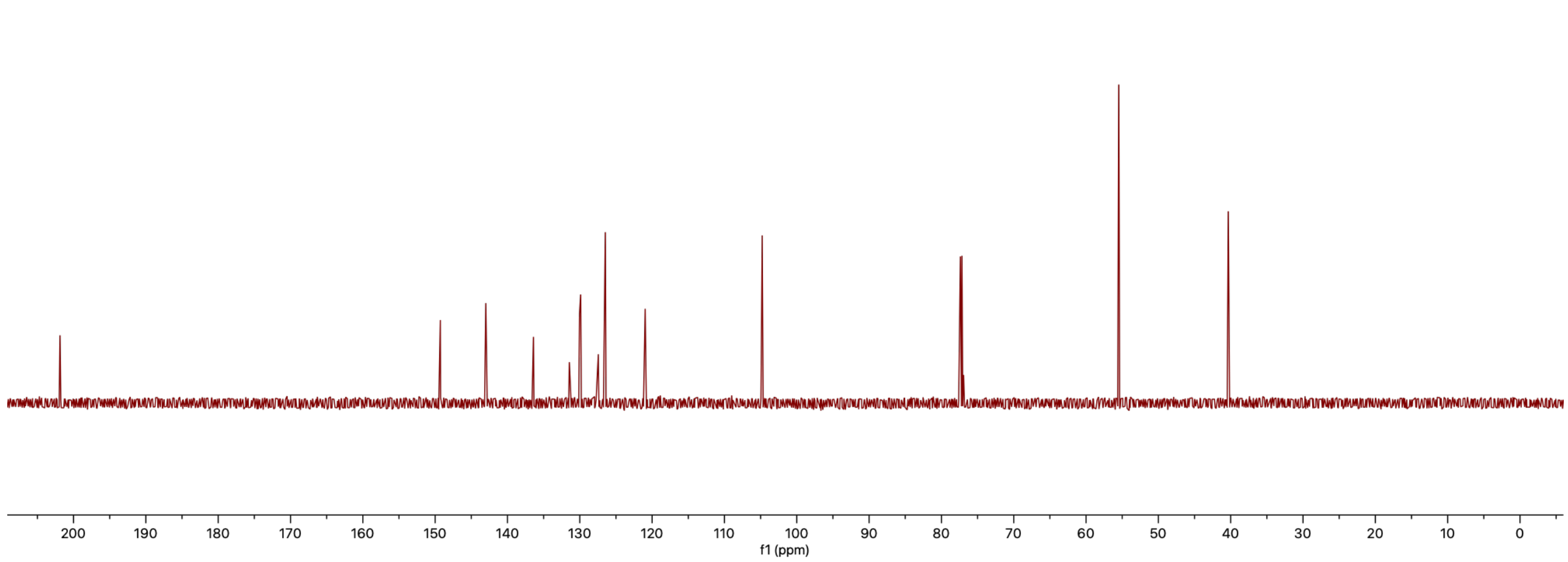

$^{13}C\{^{1}H\}$ NMR Spectrum (151 MHz, $CDCl_3$) of **14**

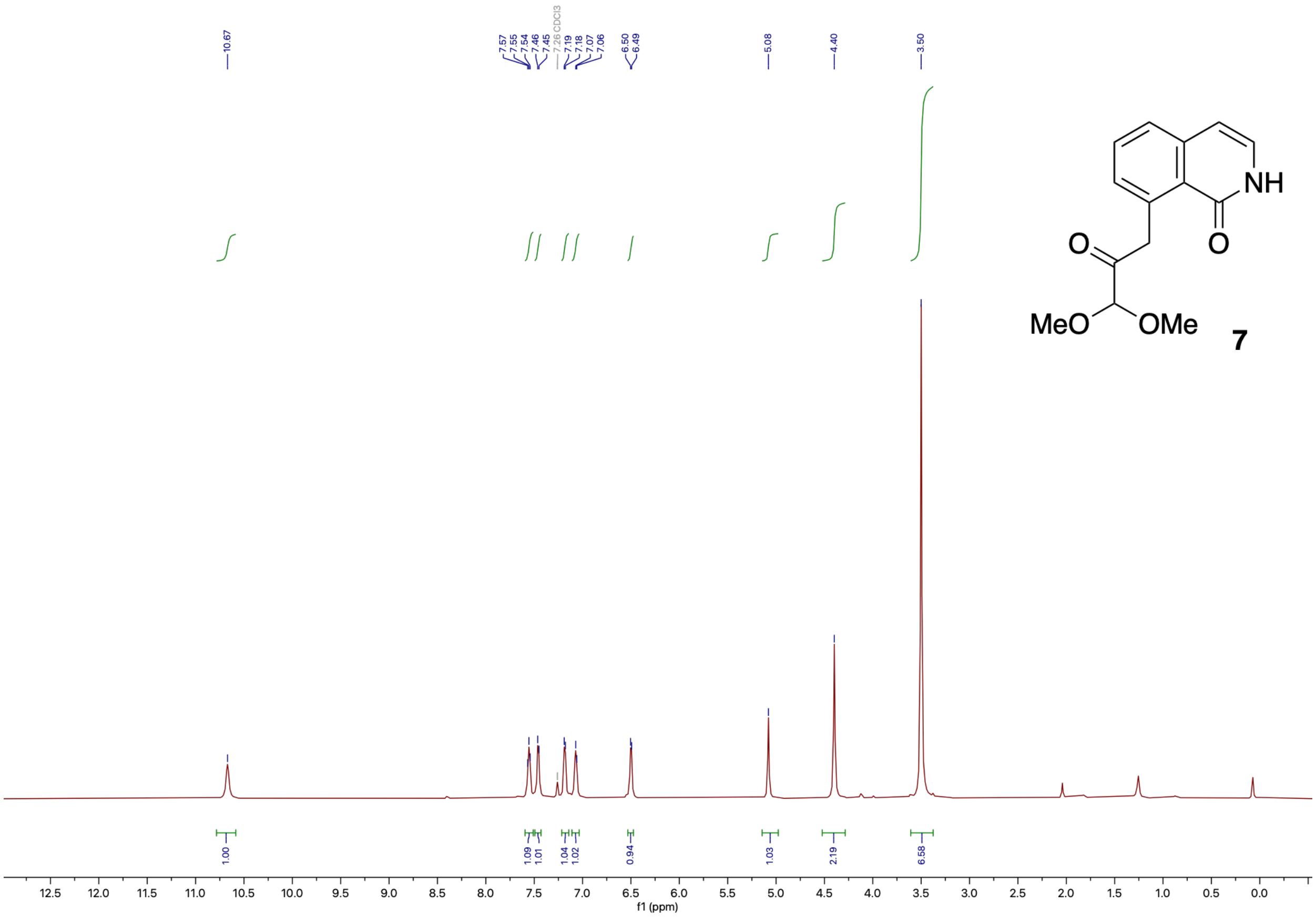

^{1}H NMR Spectrum (600 MHz, $CDCl_3$) of **7**

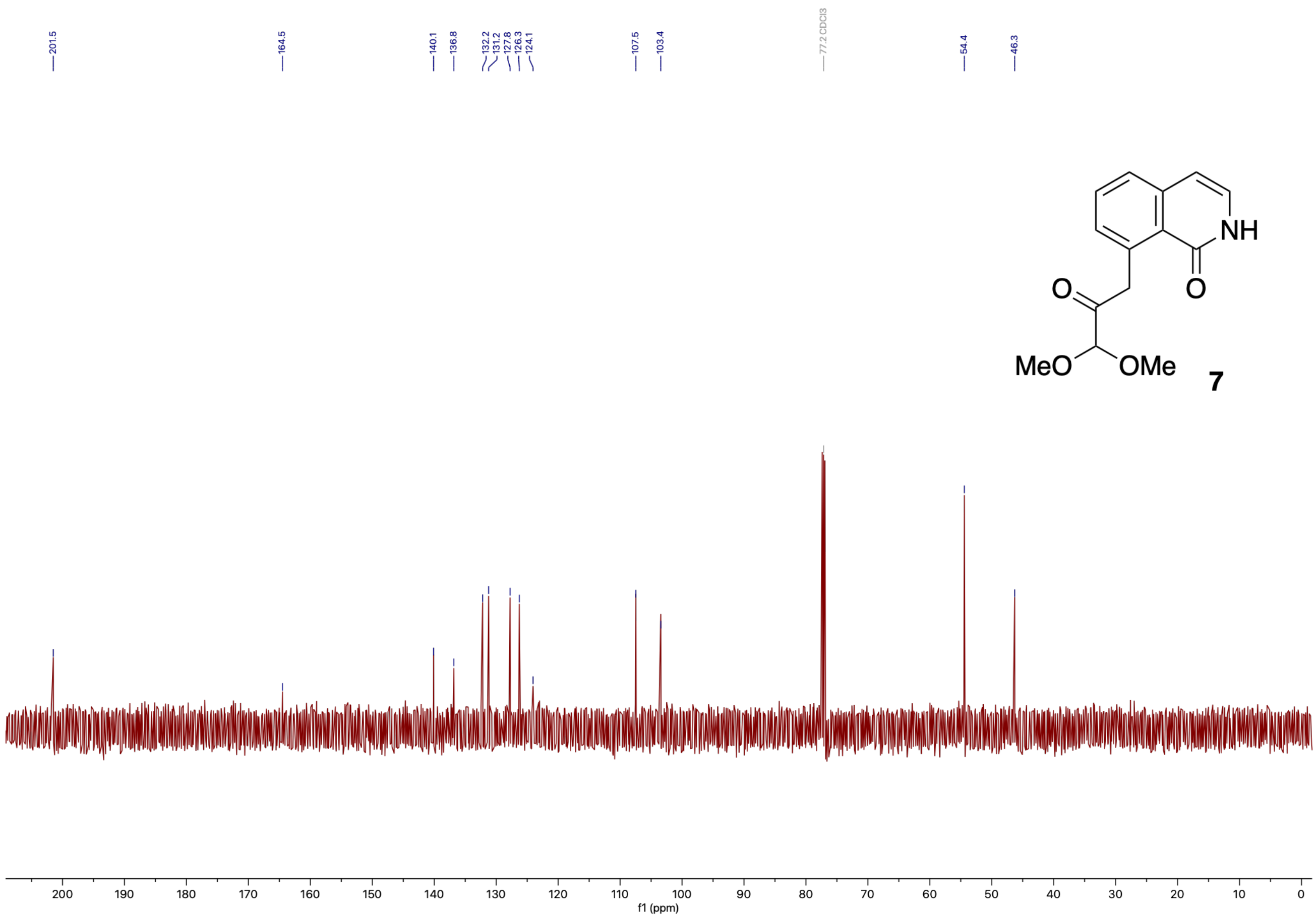

$^{13}C\{^{1}H\}$ NMR Spectrum (151 MHz, $CDCl_3$) of **7**

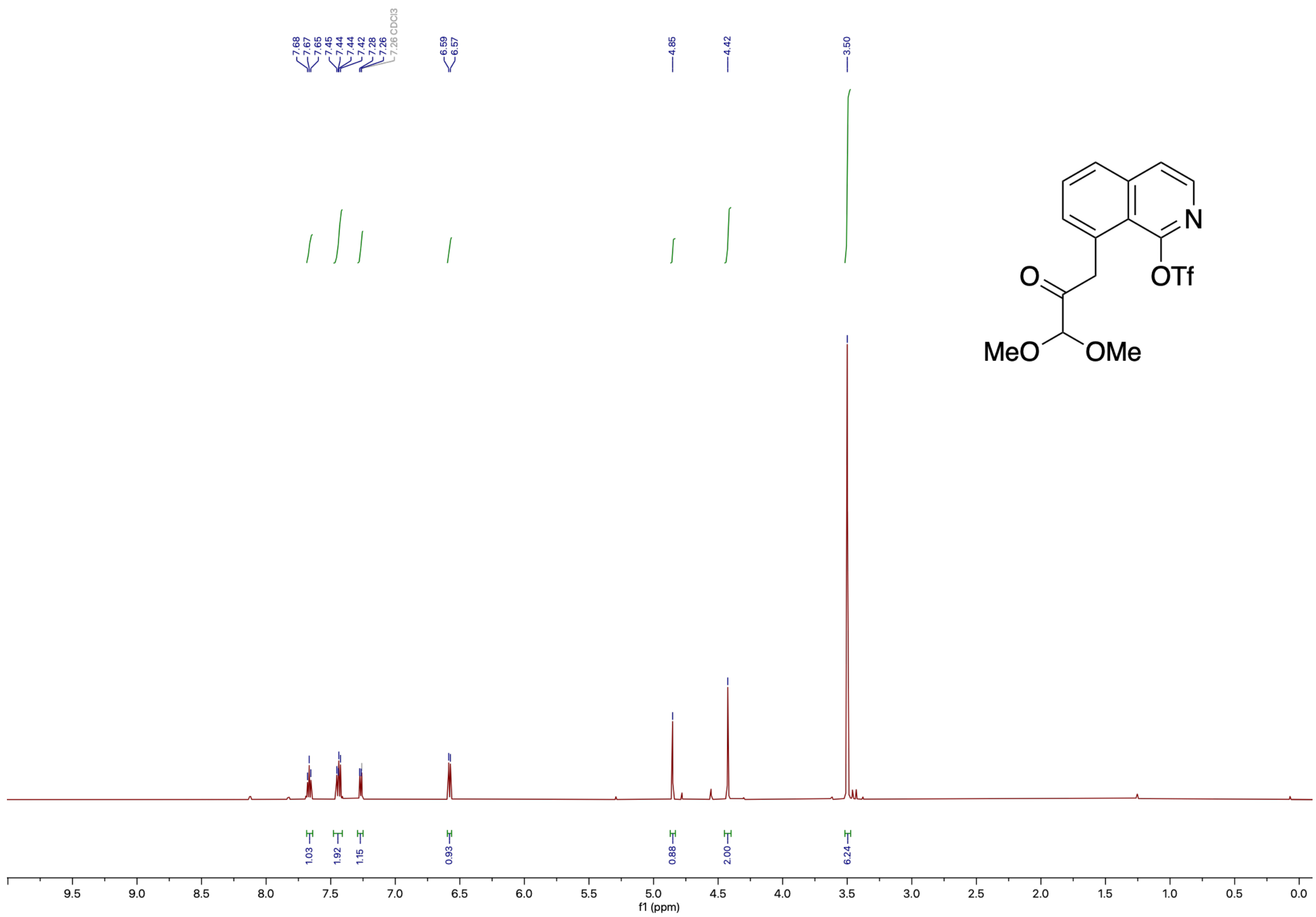

^{1}H NMR Spectrum (600 MHz, $CDCl_3$) of Triflate

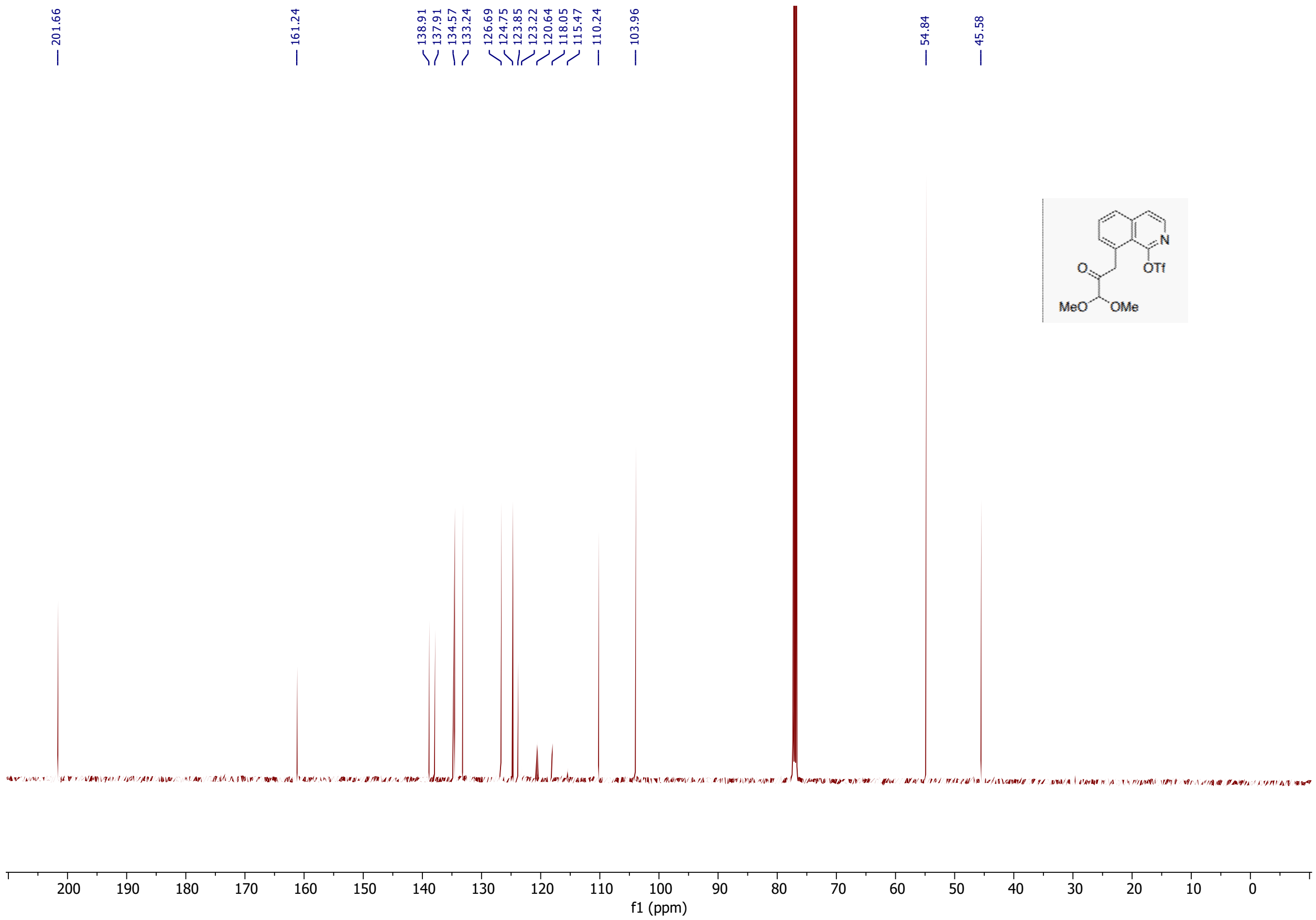

$^{13}C\{^{1}H\}$ NMR Spectrum (125 MHz, $CDCl_3$) of Triflate

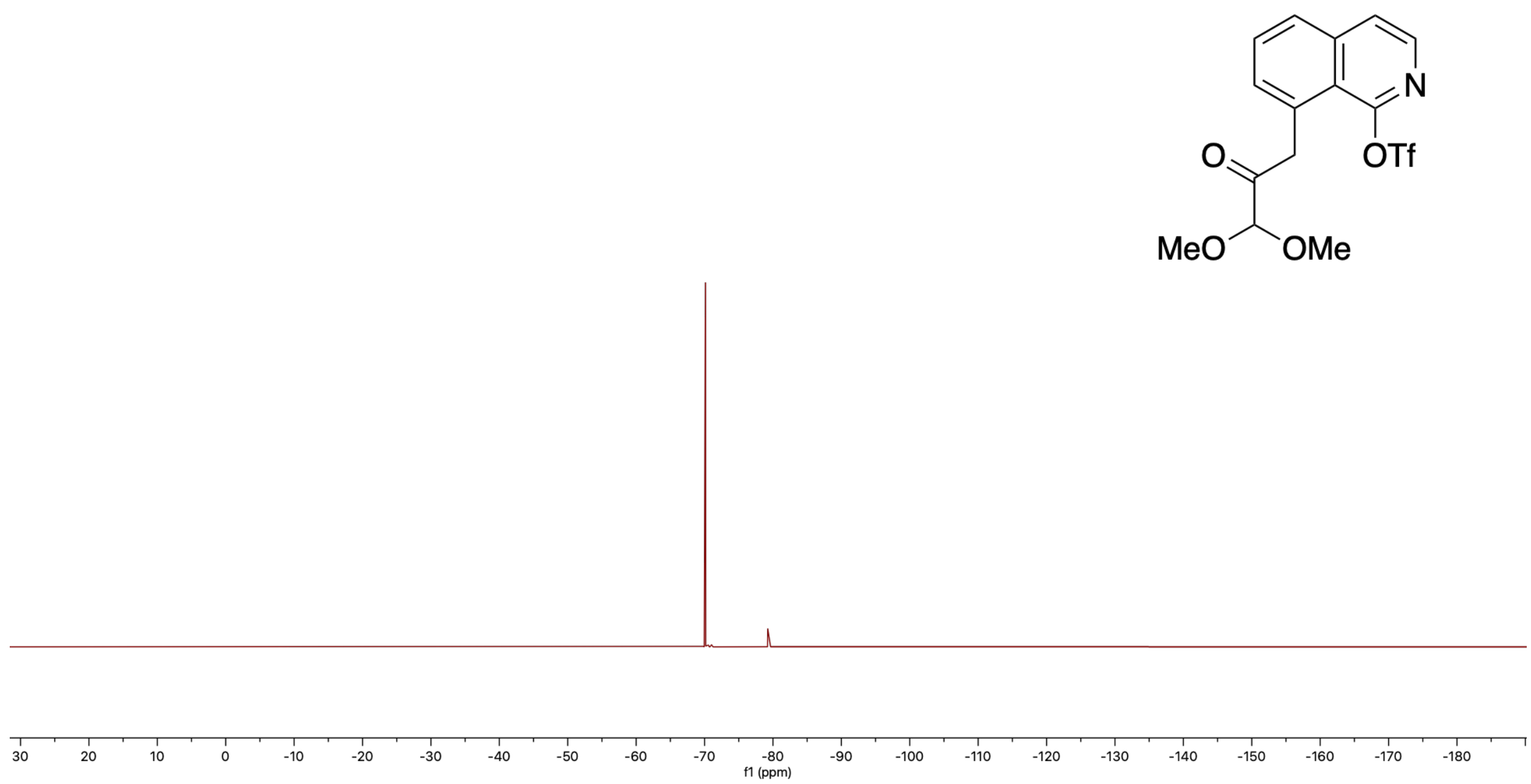

^{19}F NMR Spectrum (565 MHz, $CDCl_3$) of Triflate

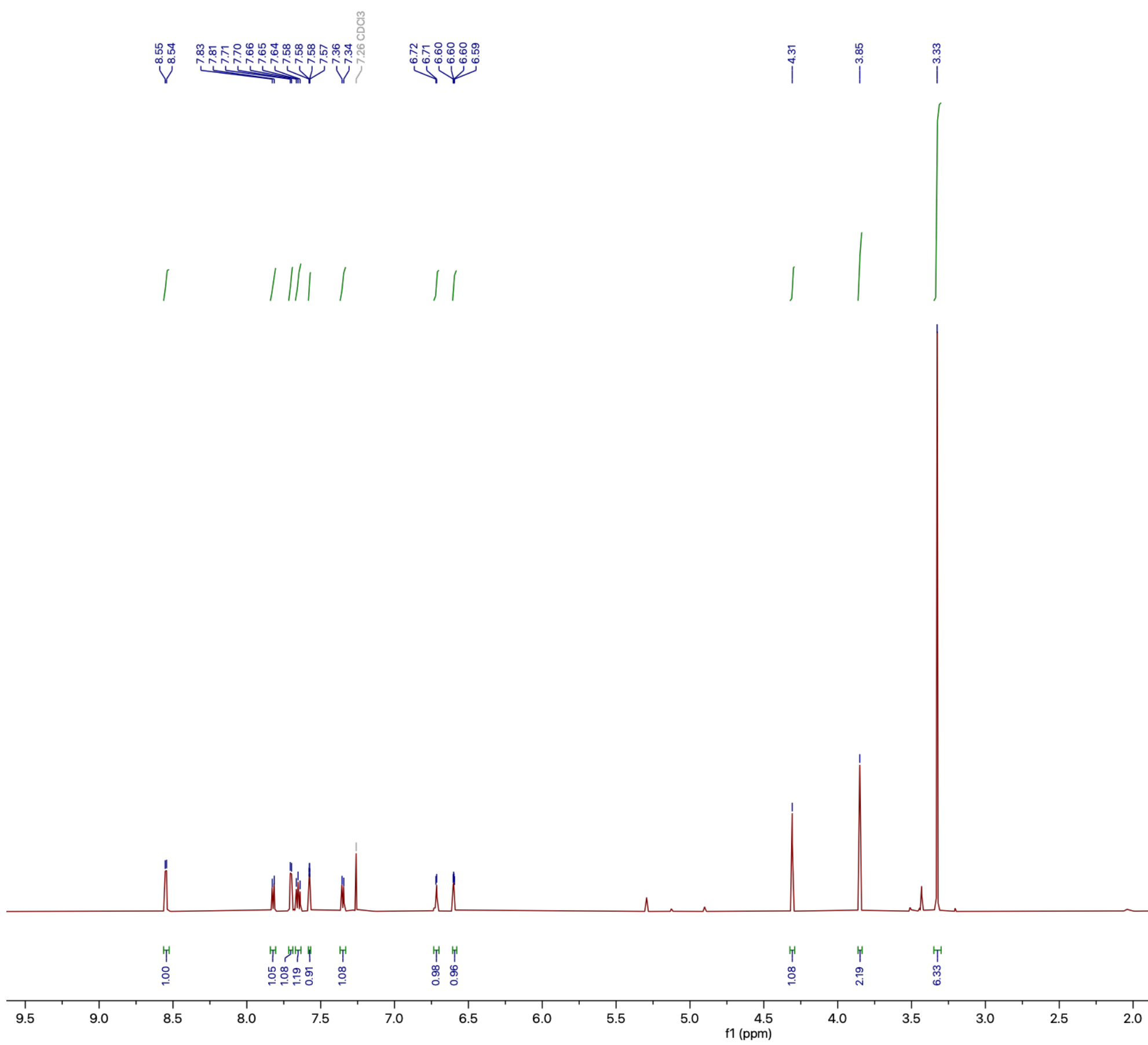

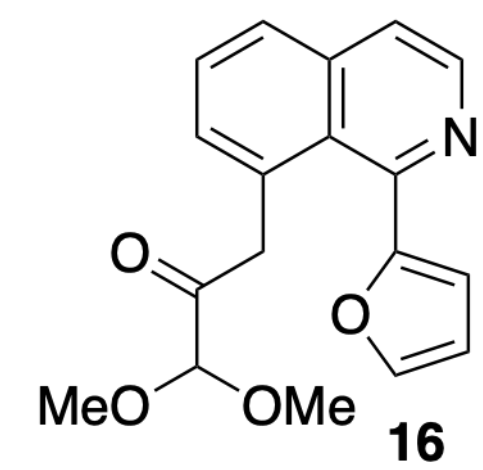

16

^{1}H NMR Spectrum (600 MHz, $CDCl_3$) of **16**

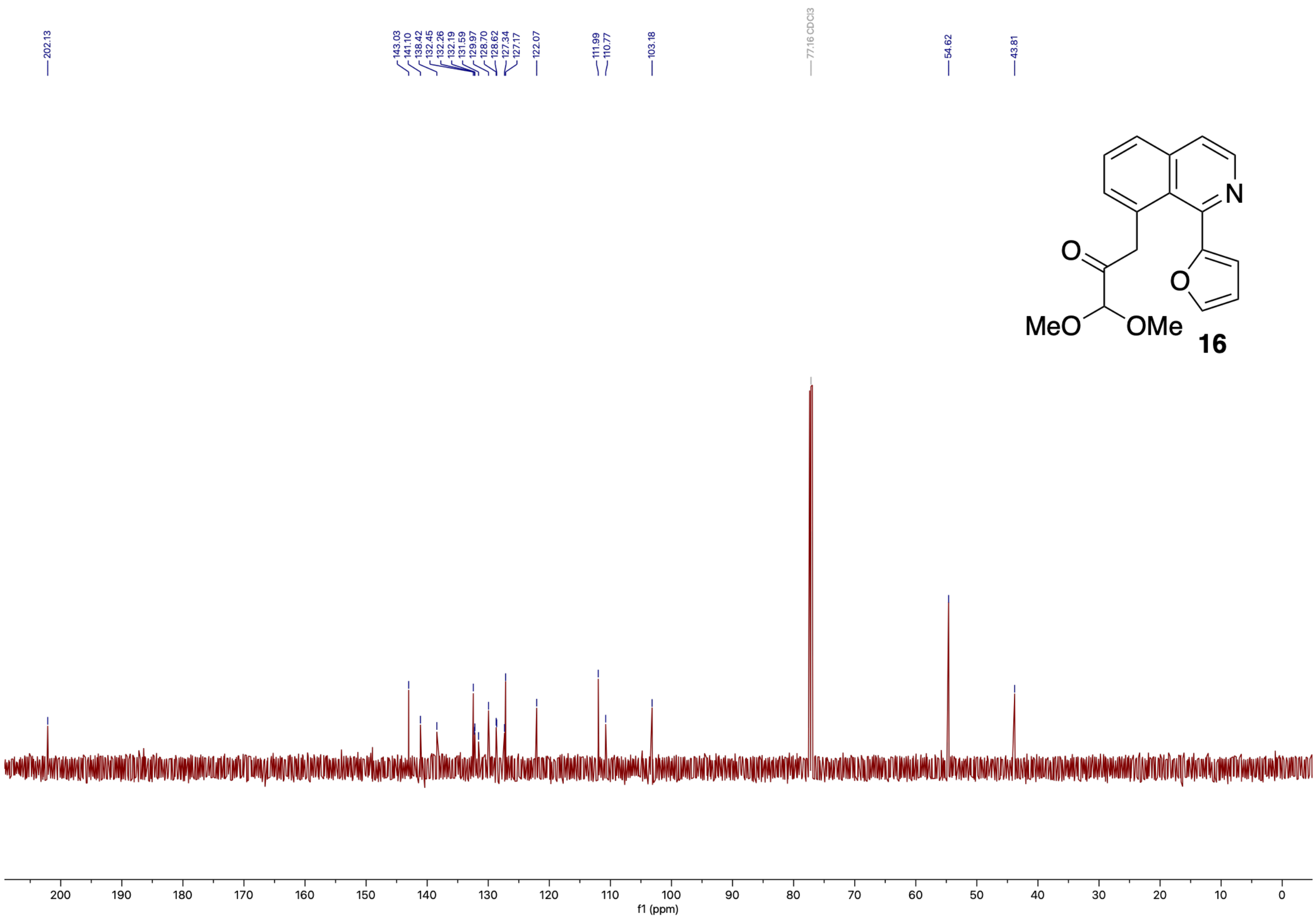

$^{13}C\{^{1}H\}$ NMR Spectrum (151 MHz, $CDCl_3$) of **16**

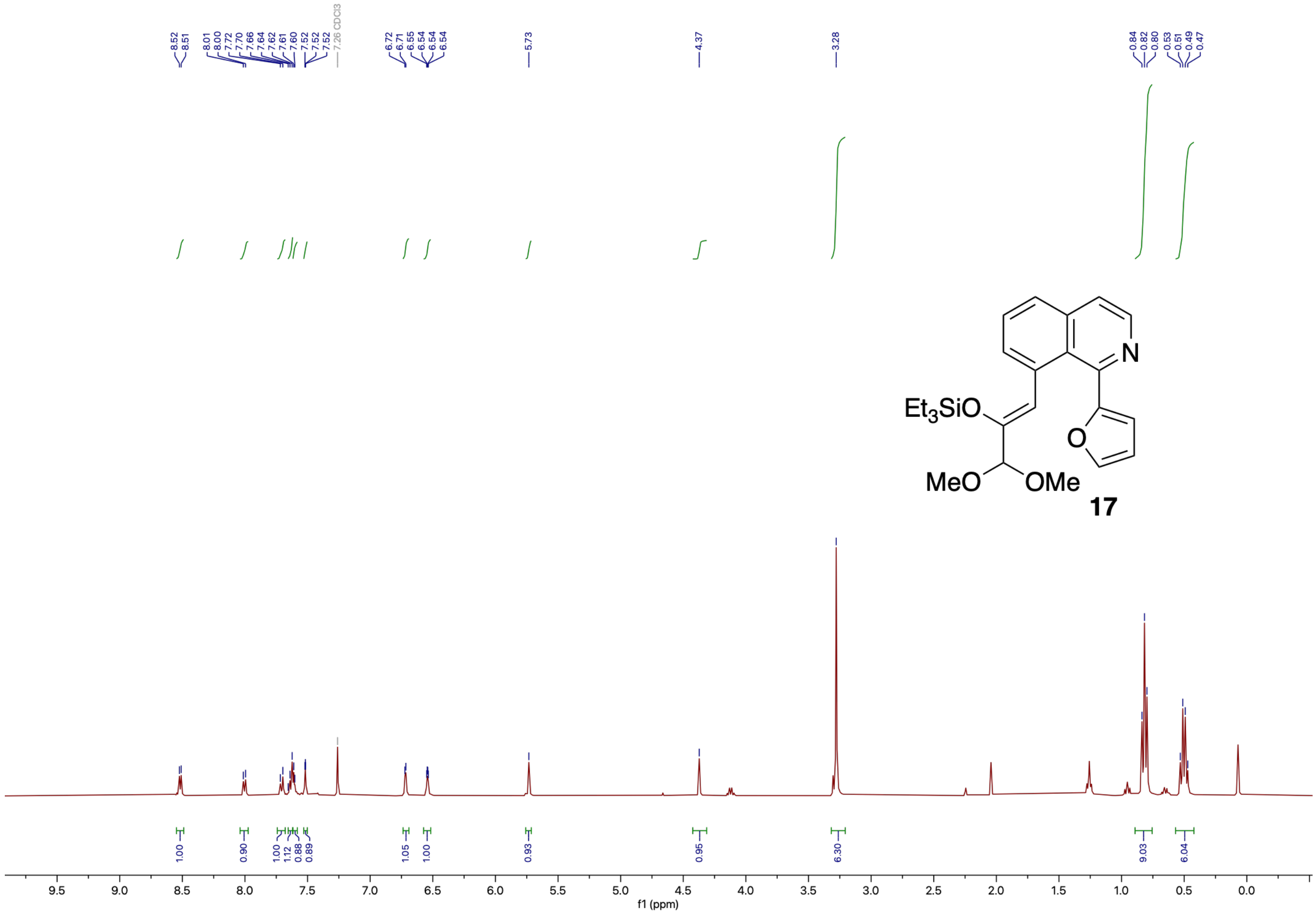

^{1}H NMR Spectrum (400 MHz, $CDCl_3$) of **17**

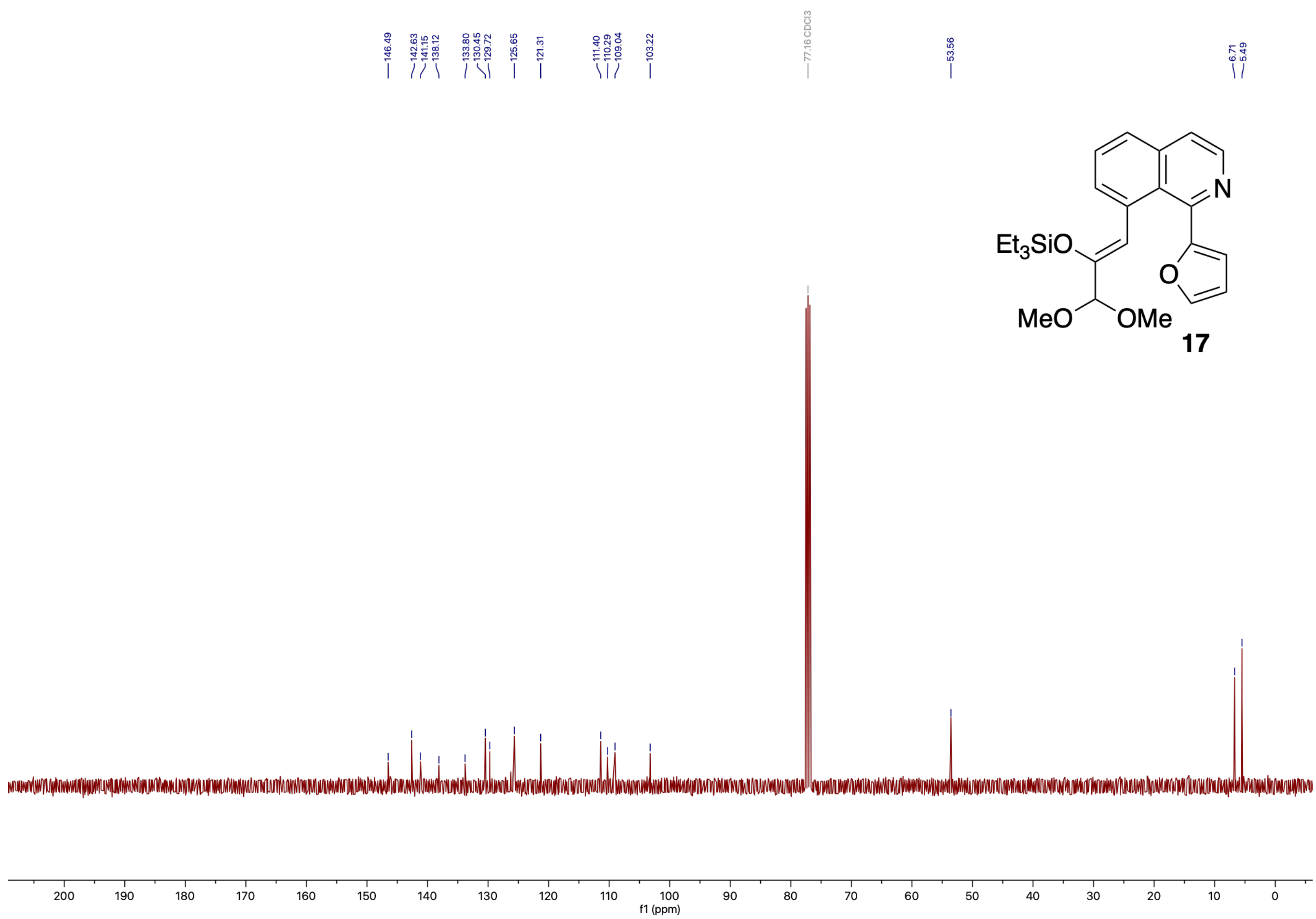

$^{13}C\{^{1}H\}$ NMR Spectrum (101 MHz, $CDCl_3$) of **17**

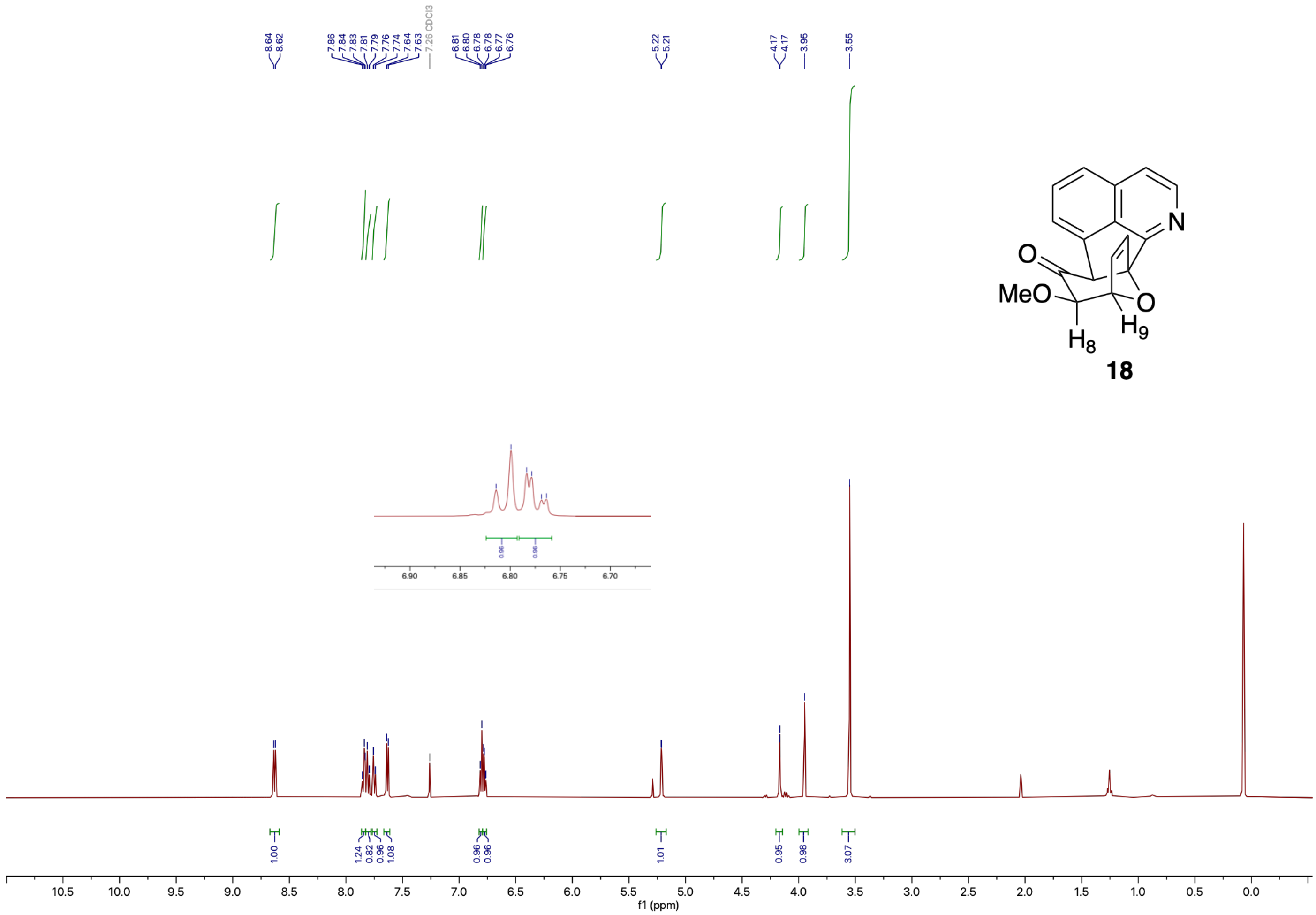

18

^{1}H NMR Spectrum (400 MHz, CDCl$_3$) of **18**

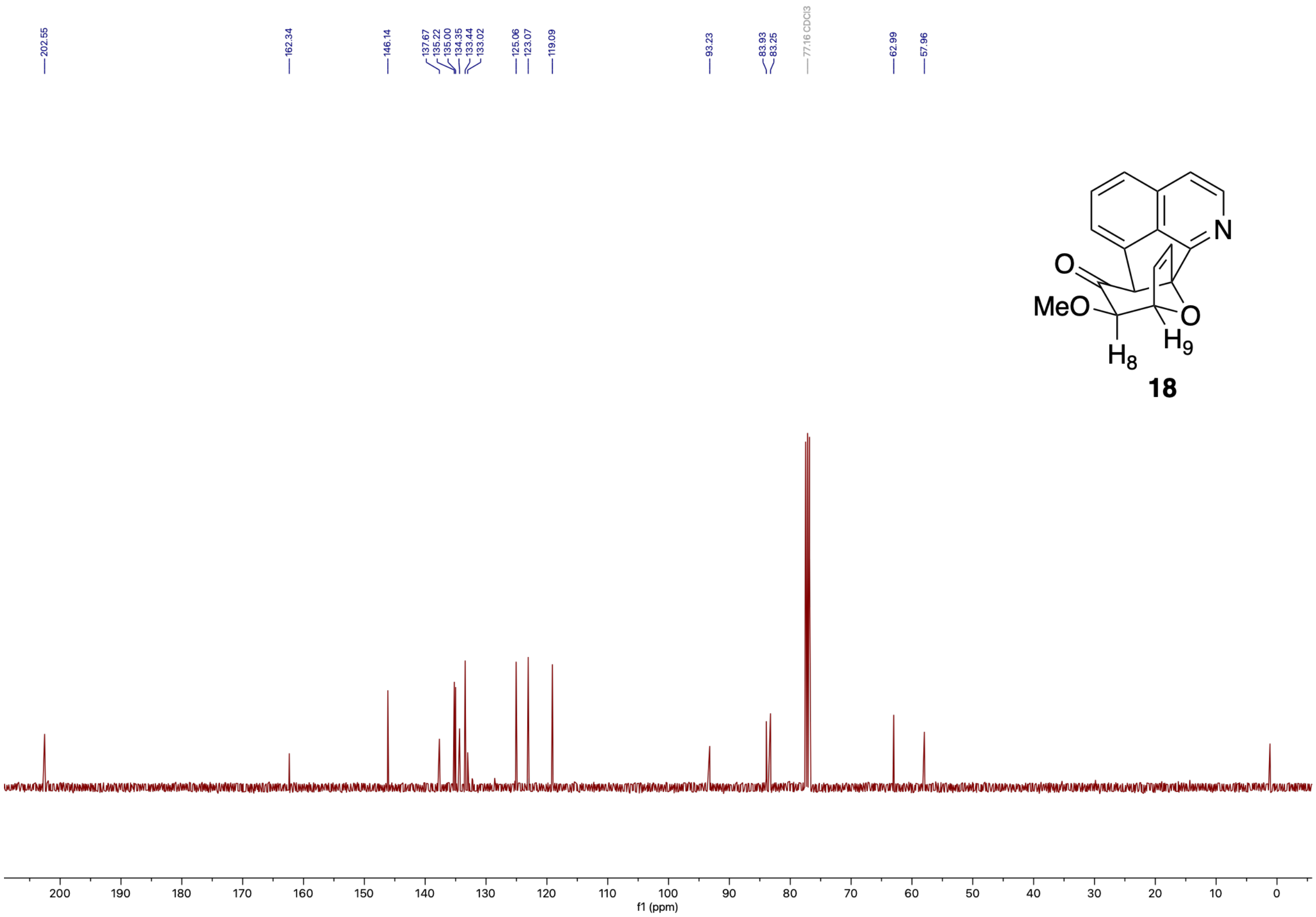

18

$^{13}C\{^{1}H\}$ NMR Spectrum (101 MHz, $CDCl_3$) of **18**

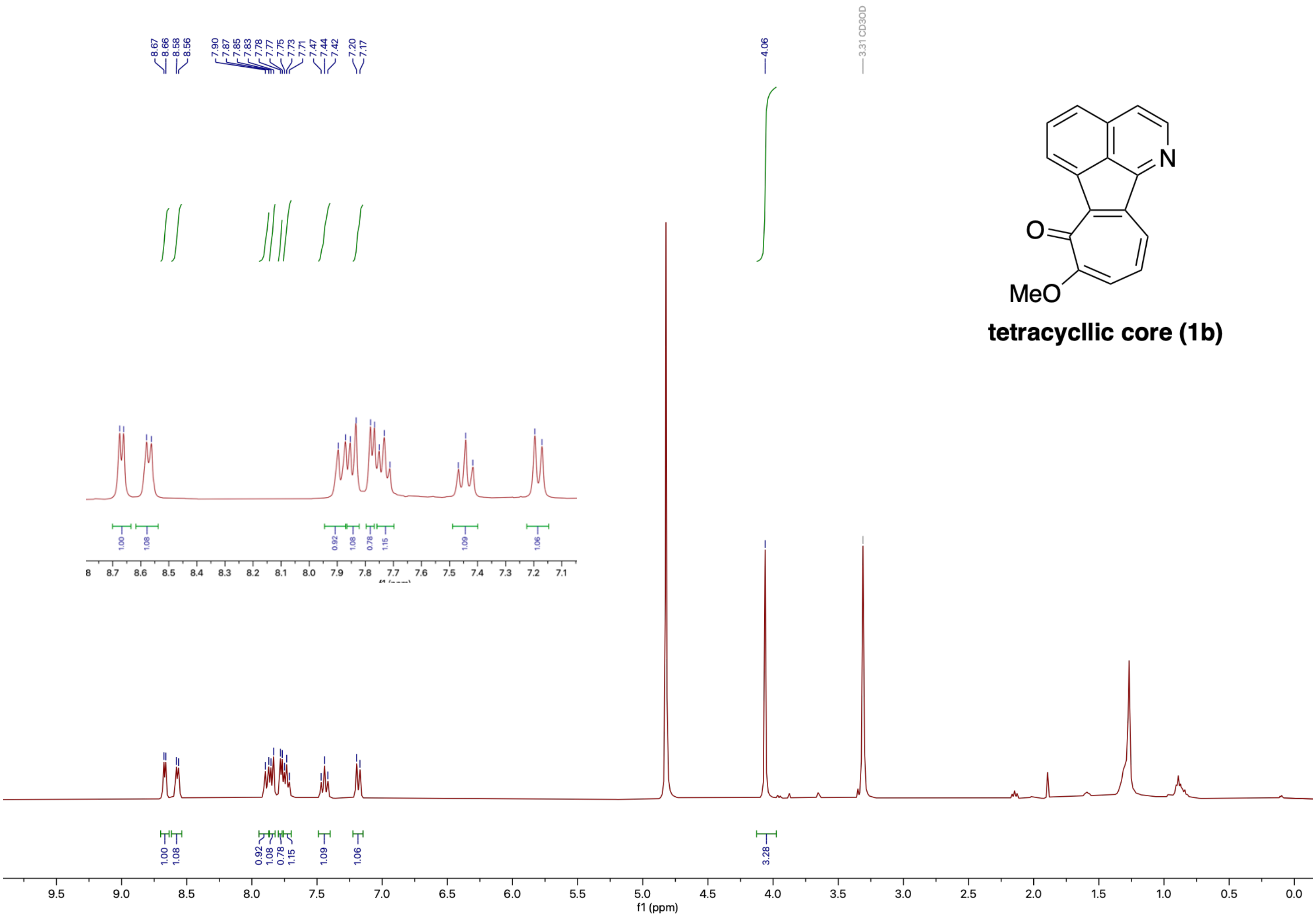

^{1}H NMR Spectrum (400 MHz, CD_3OD) of **1b**

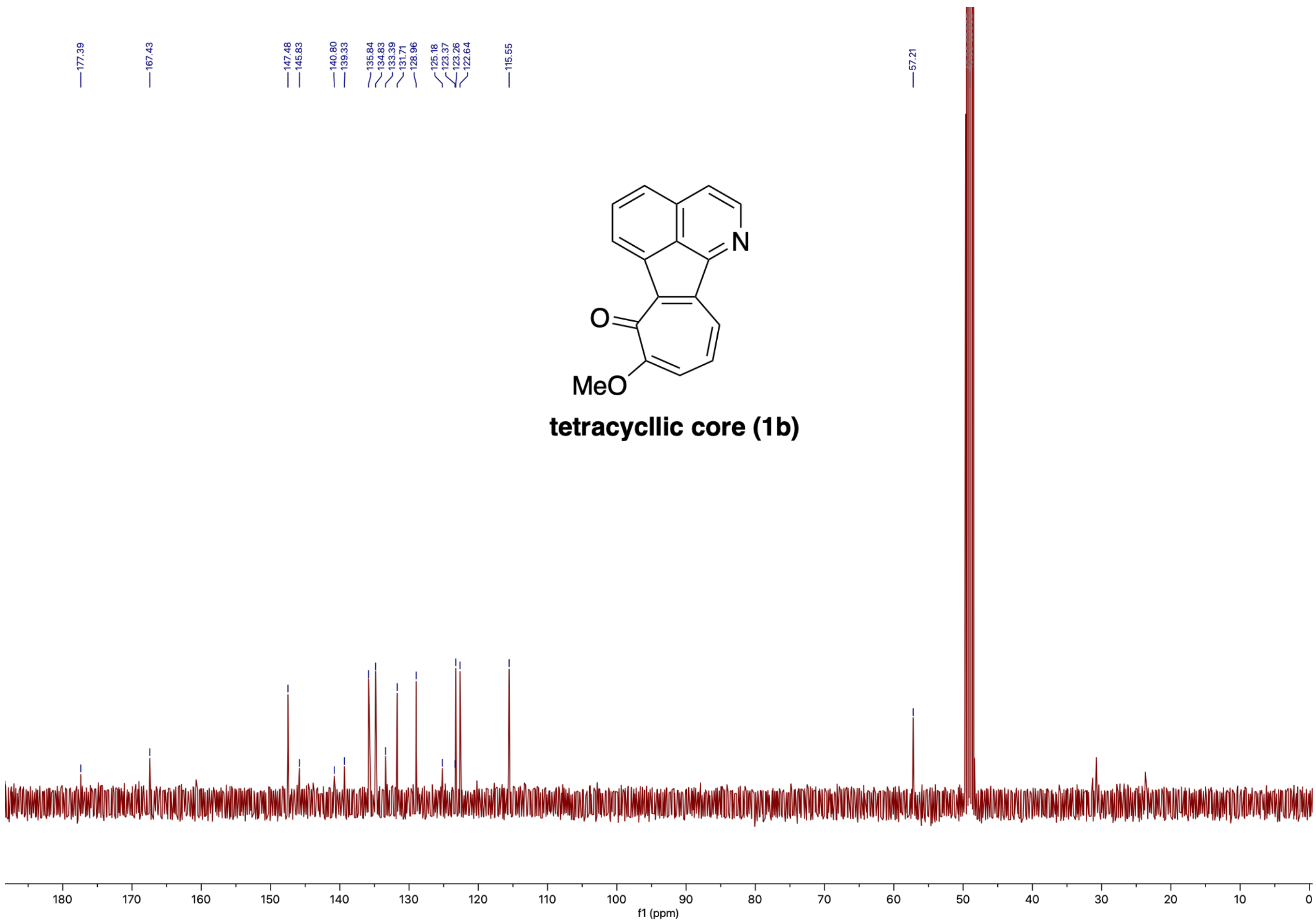

$^{13}C\{^{1}H\}$ NMR Spectrum (101 MHz, CD_3OD) of **1b**
